# Highly reproductive *Escherichia coli* cells with no specific assignment to the UAG codon

**DOI:** 10.1038/srep09699

**Published:** 2015-05-18

**Authors:** Takahito Mukai, Hiroko Hoshi, Kazumasa Ohtake, Mihoko Takahashi, Atsushi Yamaguchi, Akiko Hayashi, Shigeyuki Yokoyama, Kensaku Sakamoto

**Affiliations:** 1 Division of Structural and Synthetic Biology, RIKEN Center for Life Science Technologies, 1-7-22 Suehiro-cho, Tsurumi, Yokohama 230-0045, Japan; 2 RIKEN Systems and Structural Biology Center, 1-7-22 Suehiro-cho, Tsurumi, Yokohama 230-0045, Japan; 3 RIKEN Structural Biology Laboratory, 1-7-22 Suehiro-cho, Tsurumi, Yokohama 230-0045, Japan

## Abstract

*Escherichia coli* is a widely used host organism for recombinant technology, and the bacterial incorporation of non-natural amino acids promises the efficient synthesis of proteins with novel structures and properties. In the present study, we developed *E. coli* strains in which the UAG codon was reserved for non-natural amino acids, without compromising the reproductive strength of the host cells. Ninety-five of the 273 UAG stop codons were replaced synonymously in the genome of *E. coli* BL21(DE3), by exploiting the oligonucleotide-mediated base-mismatch-repair mechanism. This genomic modification allowed the safe elimination of the UAG-recognizing cellular component (RF-1), thus leaving the remaining 178 UAG codons with no specific molecule recognizing them. The resulting strain B-95.ΔA grew as vigorously as BL21(DE3) in rich medium at 25–42°C, and its derivative B-95.ΔAΔ*fabR* was better adapted to low temperatures and minimal media than B-95.ΔA. UAG was reassigned to synthetic amino acids by expressing the specific pairs of UAG-reading tRNA and aminoacyl-tRNA synthetase. Due to the preserved growth vigor, the B-95.ΔA strains showed superior productivities for hirudin molecules sulfonated on a particular tyrosine residue, and the Fab fragments of Herceptin containing multiple azido groups.

E*scherichia coli* is widely used for the large-scale production of industrially valuable recombinant proteins such as biopharmaceuticals and biocatalysts^1^,^2^,^3^. Since proteins are biosynthesized according to the genetic code of the host cells, the dependence on the natural code limits the recombinant technology to the standard 20 amino acids repertoire. The efforts to enhance the utility of the bacterial host have included the engineering of artificial codes encompassing the synthetic diversity of amino acids. The redundant assignments of the 64 codons to 20 amino acids and translation stop provide an opportunity for allocating degenerate codons to novel amino acids. In recent studies^4^,^5^,^6^,^7^,^8^,^9^,^10^, release factor 1 (RF-1), the cellular component recognizing the UAG triplet as a translation stop^11^, was eliminated from *E. coli*, and UAG was thus redefined as a sense codon specific for synthetic amino acids. This achievement fundamentally changed the methods of incorporating the synthetic components into proteins, with a nearly 100% incorporation efficiency. However, the RF-1 elimination severely affected the growth of *E. coli*, and the consequent reduction in the overall protein yield muted the impact of the codon redefinition.

In 1968, F. C. Crick argued that, with each of the codons occurring multiple times in the genome, a change in the meaning of any codon would destroy the proteome and thus be lethal^12^. Codon redefinition involves two distinct events: the elimination of the cellular component recognizing the codon, and the ensuing emergence of the molecular machinery to decode it with a different meaning. Recent studies^4^,^5^,^6^,^7^,^8^,^9^,^10^ have demonstrated that the RF-1 knockout, as the first step of UAG redefinition, is not lethal when certain conditions are met. Unfortunately, the serious effects on the bacterial growth still could not be circumvented. Since the translation of open reading frames (ORFs) ending with TAG is vulnerable to the absence of RF-1, in theory, as many UAG codons as possible should be replaced with other stop codons in the genome. However, there are many overlaps between ORFs or functional regions in the *E. coli* genome, and the replacement of TAG at the end of an ORF could be undesirable with respect to the overlapping gene.

In the present study, we replaced a selected set of UAG codons, and thus succeeded in preserving the reproductive strength of the RF-1-free bacteria. UAG was then safely reassigned to synthetic amino acids, to produce structurally modified proteins in a scalable manner. Thus, the artificial codes will facilitate the efforts to develop more useful and versatile protein variants, by combining the natural and non-natural diversity of amino acids.

## Results

### Strategy for modifying the genomic usage of UAG

According to the latest gene annotation, 18% of the ORFs overlap with another in the *E. coli* K-12 genome^13^. Therefore, the replacement of TAG at the end of an ORF from such a pair would cause a base substitution within the other ORF. Thus, the simple replacement of all of the UAG codons will alter the proteome. Taken together with the possible overlaps between ORFs and regulatory non-coding sequences, it has been suggested that one of the three stop codons is selected at the end of each of many genes in the genome^14^. On top of that, a large-scale engineering such as the total elimination of a codon could cause the accumulation of off-target mutations in the genome. These factors probably caused the adverse effects of the previous replacement of the 321 UAG codons in the *E. coli* genome; the resulting cells suffered a considerable reduction in the growth rate (a 60% increase in the doubling time), and accumulated 355 off-target mutations being accumulated^10^.

In constrast, RF-1 was reportedly knocked out without any modification to the UAG usage in *E. coli* B strains^8^, partly due to the inherent ability of RF-2 to barely recognize UAG and the five-fold higher activity of RF-2 from the B strains than that of RF-2 from K-12^15^. The poor growth of the engineered RF-1-free B strains confirmed the necessity of some modification to the genomic usage of UAG. However, certain mutations in *prfB*, which encodes RF-2, reportedly facilitated the UAG recognition by RF-2 and suppressed the adverse effects from the RF-1 deficiency^7^,^16^,^17^. In this case, the RF-2 variants efficiently recognized all three of the stop codons, and thus maintained the status of UAG as a translation stop.

According to the *E. coli* genome databases described in the “Methods”, seven of the ~300 ORFs ending with TAG are essential, while ~90 other genes are important for supporting vigorous growth. We previously showed that RF-1 can be knocked out, provided that TAG is replaced at the ends of the seven essential ORFs, and a UAG-reading tRNA is expressed in the cell^4^,^6^,^17^. The TAG replacement secured the expression of the essential genes, whereas the UAG-reading tRNA supported the low-level expressions of the genes still ending with TAG; a decrease in the expression level of a representative gene was observed, together with an extended C-terminus of its protein product due to UAG readthrough^17^. The growth rate of the engineered *E. coli*, called RFzero, varied depending on the identity of the amino acid assigned to UAG, and it grew well with 3-iodo-l-tyrosine and *N*^ε^-allyloxycarbonyl-l-lysine^6^. We conceived that additional UAG replacements at the ends of the important genes would enhance the growth of RFzero, and reduce the dependence on the identity of the UAG-encoded amino acid.

### Synonymous replacements of 95 UAG codons in the *E. coli* BL21(DE3) genome

**Figure 1:**
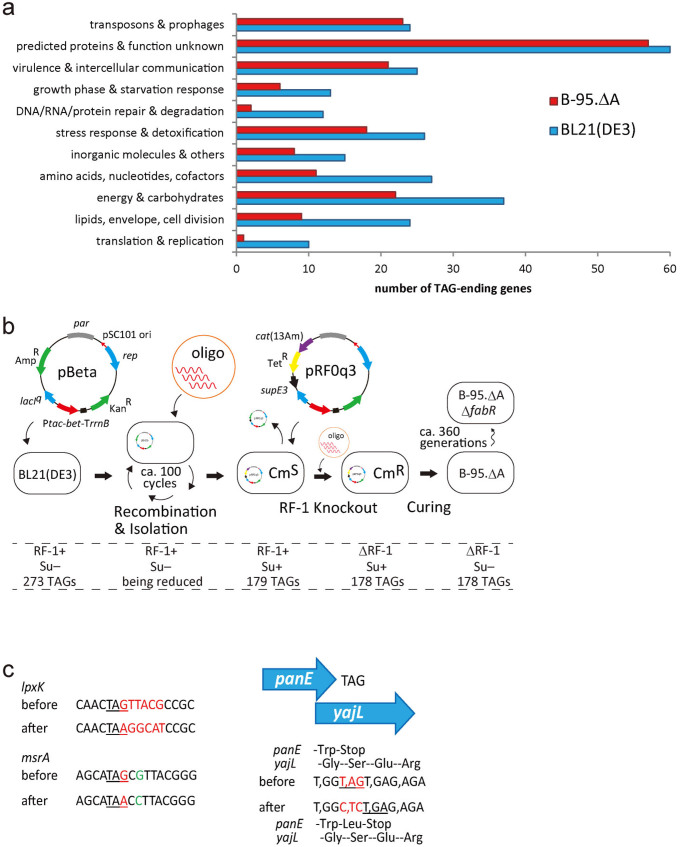
Engineering of B-95.ΔA. (a) The numbers of TAG-ending genes in BL21(DE3) (blue bars) and B-95.ΔA (red bars) are shown for each of the indicated categories. (b) The entire scheme of the present engineering. Ninety-five of the 273 UAG codons were successively replaced in the *E. coli* BL21(DE3) chromosome (left end), by the cycles of the oligonucleotide-mediated mutagenesis and the isolation of properly engineered clones. RF-1 was finally knocked out to create the strain B-95.ΔA (right end). Suppression of a *cat* amber mutant was exploited to check the RF-1 knockout. B-95.ΔAΔ*fabR* was spontaneously derived from B-95.ΔA. “Su” represents suppressor tRNA. “Cm^S^” and “Cm^R^” represent sensitivity and resistance, respectively, to chloramphenicol. “pBeta” and “pRF0q3” represent plasmids. (c) Representative oligonucleotide-mediated base substitutions. Stop codons are underlined. Changed bases are indicated in red, except for the G-to-C substitution in *msrA* (in green). The commas are inserted to indicate the reading frames within *yajL*.

We selected BL21(DE3) as the starting strain for engineering, because it is widely used for the large-scale preparation of recombinant proteins. BL21(DE3) has 273 genes ending with TAG (Supplementary Table 1), and 95 of them are important for bacterial growth, including the seven essential genes (*coaD*, *hda*, *hemA*, *mreC*, *murF*, *lolA*, and *lpxK*). TAG was to be replaced with TAA or TGA at the ends of these 95 genes, to secure their expression. Figure 1a shows the number of TAG-ending genes in each category after the planned engineering, in comparison with the original number of TAG-ending genes in BL21(DE3). Thus, TAG was replaced at the ends of most of the genes responsible for the maintenance of DNA, RNA, and proteins, and at the ends of half of the house-keeping genes involved in metabolism, energy regeneration, cell division, and responses to starvation and other stresses. The entire engineering was performed by sequential applications of oligonucleotide-mediated recombination based on Court's method^18^. This method exploits the fact that the methyl-directed repair mechanism can only inefficiently repair the base mismatches occurring in particular sequences. We applied it in a non-Δ*mutS* genetic background, in order to suppress the occurrence of spontaneous mutations, whereas the total TAG elimination previously utilized Δ*mutS* strains to facilitate the replacement of TAG^10^. Base changes were checked at each round of the application of the method, in order to isolate the cells with the intended modifications. The entire scheme of the chromosomal engineering is depicted in Figure 1b, together with illustrations of the particular TAG replacements at the ends of the *lpxK* and *msrA* ORFs (Fig. 1c). The base sequences encompassing the target UAG codons are listed in Supplementary Table 2. In most cases, the TAG replacement was facilitated by the presence of successive mismatches over 4–5 nucleotides, immediately downstream of the UAG codon. Additionally, we tried to synonymously replace certain AGG arginine codons (also listed in Supplementary Table 2), to test the feasibility of efficient codon replacement in the middle of an ORF by the employed method.

A representative TAG replacement involved in overlapping ORFs is illustrated in Figure 1c, where the ORF of *panE* runs into that of *yajL*, a gene involved in translation accuracy and oxidative stress response^19^. A simple TAG replacement at the end of *panE* would change a serine codon to an asparagine or aspartate codon within the *yajL* ORF. To avoid this, we added a leucine codon at the end of *panE* and then changed TAG to TGA. This modification does not change the amino-acid sequence of *yajL*, while minimal influence on the activity of the *panE* product was expected. In the other cases, 6–11 nucleotides were inserted just after TAG at the ends of the *yajL*, *ytfJ*, *hypB*, *tatD*, and *ycbX* ORFs, to separate the overlapping ORFs from each other and safely replace the target UAG codons. Since only the TAG at the end of the *ycaI* ORF could not be replaced in this manner, we deleted 5 bases within this ORF to create an in-frame UAA codon, so that the translation of *ycaI* terminates at this UAA codon much earlier than the regular stop position, and the overlapping ORFs were thus separated from each other.

With all of these modifications to the BL21(DE3) chromosome, TAG was replaced with TAA or TGA at the ends of the 95 genes minus *hemA*, and the resulting strain was designated as B-94. Then, we disrupted the *prfA* gene encoding RF-1, and replaced TAG at the end of *hemA* simultaneously. The successful *prfA* knockout indicated that the expression of UAG-reading tRNA is no longer necessary after the 95 TAG replacements, probably because the engineered 95 genes include most of the TAG-ending genes required to support bacterial growth. However, the tRNA expression in RFzero cells is required to allow the expression of such genes involved in cell growth. The sequence analysis of the whole chromosome of the final strain, B-95.ΔA, confirmed the absence of *prfA*, together with the occurrence of all of the intended 95 mutations. Spontaneous mutations were detected at only 9 loci. Three of them are located in the coding sequences of *cdaR*, *yfbS*, and *tolQ* and do not change the identity of the amino acids (Table 1). Two are deletions within the ORFs of the dispensable *dcp* and *yjhB* genes, while the other four probably cause no serious effect. The mutations that occurred at *plsX* and *serA* in B-94 were repaired before the *prfA* knockout.

### Characterization of the growth of B-94 and B-95.ΔA and isolation of a derivative of B-95.ΔA adapted to minimal media

**Figure 2:**
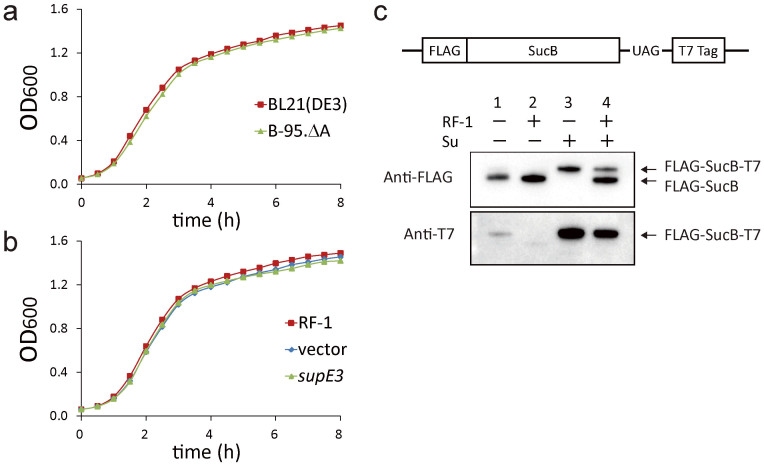
Characterization of B-95.ΔA in comparison with the parent strains BL21(DE3) and B-94. (a) The growth profiles of BL21(DE3) (red line) and B-95.ΔA (green line) in LB medium at 37°C. (b) The growth profiles of B-95.ΔA transformed with pAp15, a control plasmid (blue line), the pAp15-RF1 plasmid expressing RF-1 (red line), and pAp15-supE3 expressing the SupE3 tRNA (green line) in LB with kanamycin (15 μg/ml) at 37°C. (c) Expression of a SucB variant tagged at both termini in B-95.ΔA (lanes 1–3) and B-94 (lane 4). RF-1 was expressed from the plasmid pAp15-RF1 in lane 2. The SupE3 tRNA was expressed from pAp15-supE in lanes 3 and 4. The expression was analyzed by fractionation on a NuPAGE gel, followed by western blotting with the antibodies against the FLAG and T7 tags at the N- and C-termini, respectively.

At 37°C in rich Luria-Bertani (LB) medium, B-95.ΔA grew at the same rate as BL21(DE3) (Fig. 2a), and B-94 also grew as vigorously (Supplementary Fig. 1b). At a high temperature (42°C), these strains also grew at the same rate (Supplementary Fig. 1c). At lower temperatures (30 and 25°C), BL21(DE3) and B-94 grew at similar rates, whereas B-95.ΔA grew more slowly (Supplementary Fig. 1d, e). All three strains grew under anaerobic conditions in LB medium at 37°C (Supplementary Fig. 2), and also in M9 minimal media containing 1mM MgSO_4_ and 1% glucose or 2% glycerol as the carbon source (Supplementary Fig. 3a, b). Thus, B-94 can grow as vigorously as BL21(DE3) under various conditions, which indicates that the replacement of a third of the UAG codons did not impair the fitness of *E. coli*. The elimination of RF-1 from B-94, to create B-95.ΔA, only slowed the growth rate at the lower temperatures.

In the case of RFzero cells, a spontaneous mutation that created a strong amber suppressor tRNA reportedly enhanced the cell growth by facilitating the expression of TAG-ending genes^17^. We examined if the presence of UAG-reading tRNA would also enhance the growth of B-95.ΔA. The introduction of SupE3 tRNA^Gln^, an amber suppressor that efficiently recognizes UAG^17^, did not improve the growth of B-95.ΔA in either LB medium at 37°C (Fig. 2b) or the glycerol minimal medium supplemented with leucine (GMML) at 37°C (Supplementary Fig. 3d). These observations were consistent with the successful knockout of RF-1 in B-95.ΔA in the absence of UAG-reading tRNA. Thus, we expected that continuous culturing of B-95.ΔA would favor mutations that help the strain to adapt to the given environment, rather than cause trivial mutations to create amber suppressor tRNAs.

B-95.ΔA grew more slowly than B-94 in M9-glucose liquid medium (with a 1.4-fold longer doubling time) (Table 2) and on M9-glycerol agar plates (Supplementary Fig. 3b). B-95.ΔA was cultured in M9-glucose medium over 360 generations, during which the culture was diluted 30 times each by a factor of 5,000. The resulting population consisted of the same variant with a frameshift mutation in *fabR*. The deletion of this gene reportedly elevated the levels of unsaturated fatty acids in the membrane phospholipids^20^. The isolated strain, B-95.ΔAΔ*fabR*, showed improvements in the growth rate and the maximal cell density in M9-glucose medium, and these values were comparable to those of B-94 (Table 2). B-95.ΔAΔ*fabR* also grew on the M9-glycerol agar plate as vigorously as BL21(DE3) and B-94 (Supplementary Fig. 3c). Finally, we examined the growth rate of B-95.ΔAΔ*fabR* in LB at 16°C, and found that the growth rate is improved, with the maximal cell density matching that of B-94 (Table 2).

### Effects of the RF-1 knockout on the expression of genes ending with TAG

B-95.ΔA lacks any specific molecule that recognizes the UAG codon, and two-thirds of the codons are thus left in the genome with no such molecule in the cell. The proteomes of B-94 and B-95.ΔA were analyzed by two-dimensional polyacrylamide gel electrophoresis (2D-PAGE), in comparison with that of BL21(DE3). The difference between BL21(DE3) and B-94 is quite small, with 17 protein spots having different intensities (Supplementary Figs. 4, 5; Supplementary Tables 5, 6). A larger difference was found between BL21(DE3) and B-95.ΔA, with changes in 42 spots (Supplementary Figs. 4, 5; Supplementary Tables 7, 8). Some proteins were identified by MS/MS ions searches (Supplementary Table 9), revealing a reduction in the expression levels of *ybl129* and *yhbW*, which had TAG at their ends. The increased levels of *frmA* and *frmB* were interpreted as a consequence of the possible reduction in the expression of the repressor, encoded by *frmR* ending with TAG. These findings indicated that the absence of RF-1 in B-95.ΔA did not cause an extensive change in the proteome, with the observed effects confined to TAG-ending genes and related genes.

Next, we more directly analyzed the status of the UAG codon in B-95.ΔA, by examining the expression of a *sucB* variant gene with an in-frame UAG codon just before the C-terminal T7 tag. The ORF of this *sucB* variant ended with TAA, and had a FLAG tag at the N-terminus as a second tag. With this construct, the translation termination at UAG will produce a truncated protein product lacking the T7 tag (FLAG-SucB). In B-95.ΔA, the full-length product (FLAG-SucB-T7) was expressed at a marginal level (Fig. 2c, lane 1), and its synthesis was completely repressed by expressing RF-1 from a plasmid (Fig. 2c, lane 2). Interestingly, FLAG-SucB was expressed at a low level even in B-95.ΔA (Fig. 2c, lane 1), which suggested the occurrence of translation termination at UAG in the absence of RF-1. The significance of this observation will be discussed later.

The expression of the UAG-reading SupE3 tRNA in B-95.ΔA dramatically changed the expression profile of the SucB variant; only the full-length product FLAG-SucB-T7 was synthesized, whereas the truncated product FLAG-SucB was not detected (Fig. 2c, lane 3). By contrast, the truncated product was synthesized preferentially in B-94, although the SupE3 tRNA allowed the full-length molecule to be expressed to some level (Fig. 2c, lane 4). The expression level of FLAG-sucB-T7 due to the UAG readthrough was significantly higher in B-95.ΔA than B-94, which was consistent with the absence of RF-1 in B-95.ΔA. Thus, the expression of the UAG-reading tRNA in B-95.ΔA immediately caused UAG to behave like a full-fledged sense codon. It is worth noting here that specific molecular machinery, consisting of the pair of a UAG-reading tRNA and a variant aminoacyl-tRNA synthetase (aaRS), has been developed for each of more than 100 synthetic amino acids^21^,^22^.

### High productivity of B-95.ΔA for recombinant proteins with natural or non-natural modifications

Finally, we demonstrated the utility of B-95.ΔA in synthesizing therapeutic proteins containing synthetic amino acids. Hirudin, the most potent inhibitor of thrombin, is produced in a recombinant form. It is naturally sulfated on tyrosine at position 63. The specific pair of UAG-reading tRNA and aaRS for *O*-sulfo-l-tyrosine has been developed, and its incorporation in place of Tyr63 reportedly enhances the binding of sulfo-hirudin to thrombin^23^. We used B-95.ΔAΔ*fabR* as the host cells to synthesize sulfo-hirudin, because of the strain's improved fitness in minimal media. Clinically administered recombinant proteins are preferably produced by the host cells cultured in synthetic media, because unidentified ingredients from organic media, such as LB, may contaminate the recombinant proteins.

**Figure 3:**
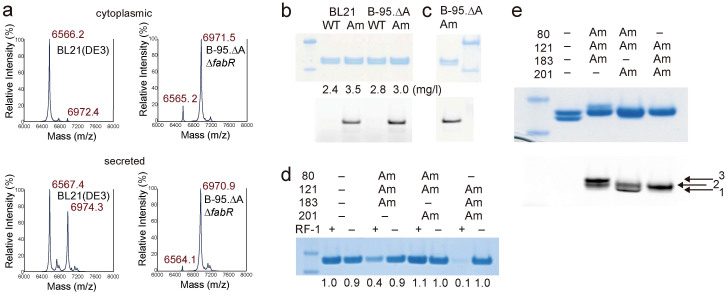
Production of hirudin and a Fab fragment containing synthetic amino acids in BL21(DE3) and B-95.ΔA. (a) MALDI-TOF analyses of the recombinant HV1 products from the gene with TAG at position 63. The products were from the growth medium (left) and the cytoplasm (right) of BL21(DE3) and B-95.ΔAΔ*fabR*. The calculated masses (m/z) of the full-length HV1 with and without *O*-sulfation are 7050.5 and 6970.5, respectively, for [M+H]^+^. Note that sulfotyrosine can easily be deacylated in this matrix. The calculated mass (m/z) of the truncated HV1 with translation terminated at UAG is 6566.1 for [M+H]^+^. (b) Fab fragments produced in BL21(DE3) and B-95.ΔA. The purified products and those subsequently labeled with a fluorescent probe were fractionated on NuPAGE gels (in the upper and lower gels, respectively). Fluorescence was detected using an LAS4010 image analyzer. The labeled products correspond to the heavy chain. “WT” and “Am” indicate the products expressed from genes with and without an in-frame TAG, respectively. The figures indicate the yields of the products (mg/l). (c) The Fab fragment from B-95.ΔA, labeled and then purified by protein-G column chromatography, was analyzed by electrophoresis on a NuPAGE gel. (d) Fab fragments containing three 4-azidophenylalanines at the positions marked with “Am” were synthesized in BL21(DE3) (“RF-1+”) and B-95.ΔA (“RF-1−”), and then were analyzed by electrophoresis on a NuPAGE gel. The figures at the bottom of the gel indicate relative yields for the variants. (e) The Fab fragments containing three 4-azidophenylalanines at the positions marked with “Am” were synthesized in B-95.ΔA and labeled, followed by a NuPAGE analysis (upper) and fluorescence detection (lower). The positions corresponding to the Fab fragments labeled at one, two, and three sites are indicated. The size markers in the right-most or left-most lanes in panels b–e indicate 20 and 30 kDa.

BL21(DE3) and B-95.ΔAΔ*fabR* were transformed with the genes coding for the sulfotyrosine-specific tRNA-aaRS pair. The tyrosine codon at position 63 was changed to UAG. Hirudin variant 1 (HV1) in the sulfated form was either expressed in the cytoplasm or secreted into the growth medium as a fusion protein with a cleavable N-terminal 6× His-SUMO tag. As for the cytoplasmic expression, similar amounts of BL21(DE3) and B-95.ΔAΔ*fabR* cells were harvested from the same culture volume, and a larger amount of HV1 was purified from BL21(DE3) than B-95.ΔAΔ*fabR*; the yields were 9.8 and 1.5 mg per liter of culture, respectively. However, mass spectrometric analyses revealed that >90% of the HV1 from BL21(DE3) was the truncated molecule with translation terminated at UAG, whereas B-95.ΔAΔ*fabR* mostly produced the full-length HV1 (Fig. 3a). Thus, incomplete translation accounted for the majority of the larger yield of HV1 from BL21(DE3). Similarly, the secretion system also produced a larger amount of HV1 from BL21(DE3) than B-95.ΔAΔ*fabR*, with the yields of 2.3 and 0.7 mg/l, respectively. Mass spectrometric analyses revealed that the HV1 molecules from BL21(DE3) were a mixture of the truncated and full-length molecules, whereas B-95.ΔAΔ*fabR* mostly secreted the full-length molecule (Fig. 3a).

Since the *O*-sulfonate group was degraded in these MS analyses, we changed the matrix, as previously reported^24^, and confirmed the sulfation of the full-length HV1 from each strain (Supplementary Fig. 6). The sulfo-HV1 prepared from B-95.ΔAΔ*fabR* exhibited the anti-thrombin activity in biochemical assays (Supplementary Fig. 7). The results clearly indicated the advantage of B-95.ΔAΔ*fabR* as a host organism, which supported the high-level expression of a homogeneous product from a gene variant with an in-frame stop codon.

Next, we used B-95.ΔA to synthesize the Fab fragments of an antibody. The incorporation of synthetic amino acids with bio-orthogonal reactive groups allows the site-selective chemical modification of proteins, with minimal effects on their functions and structures. This strategy has successfully been applied to chemically conjugate two different Fab fragments and create a bispecific Fab dimer^25^. We incorporated 4-azido-l-phenylalanine into the Fab fragment of Herceptin at specific sites using BL21(DE3) and B-95.ΔA, which were each transformed with the gene pair for the UAG-reading tRNA and aaRS specific to 4-azidophenylalanine^26^. Similar amounts of the Fab variant with an azido group at position 121, in the CH1 domain, were synthesized in (3.5 and 3.0 mg/l) in BL21(DE3) and B-95.ΔA, respectively (Fig. 3b). These yields were similar to those from the “native” Fab expressed from a gene without an in-frame TAG [2.4 and 2.8 mg/ml in BL21(DE3) and B-95.ΔA, respectively]. The incorporation of the azido group was confirmed by the chemical conjugation with Rhodamine via the Staudinger-Bertozzi reaction^27^,^28^, followed by the detection of fluorescence. Furthermore, the labeled Fab from B-95.ΔA was bound, and then eluted from a protein-G column (Fig. 3c), which indicated that the molecule retained the native structure. The affinities of the wild-type Fab and the azido-Fab for the extracellular domain (1–646 a.a.) of the ERBB2 receptor were determined to be 1.2 and 1.0 nM, respectively, which are comparable to the reported value for the Herceptin Fab fragment (~1.0 nM)^29^.

We then tried to incorporate 4-azidophenyalanine into the Fab fragment at multiple specific sites simultaneously. The Fab gene variant had TAG at three of the four positions 80, 183, 121, and 201. When BL21(DE3) was used as the host, the expression level of the gene variant varied with the combination of the incorporation sites, and the synthesis of the Fab variant with 4-azidophenyalanines at positions 121, 183, and 201 was hardly supported (Fig. 3d). On the other hand, B-95.ΔA efficiently incorporated the amino acid at any combination of three positions, and the yields of the Fab variants were almost the same as that of the “native” Fab molecule. We then found that the most efficient labeling with FITC was achieved with the combination of positions 80, 121, and 183 (Fig. 3e). Thus, when a Fab fragment was selected as a target, B-95.ΔA showed a clear advantage over BL21(DE3) in the multiple-site incorporation of a synthetic amino acid.

## Discussion

In the present study, we showed that RF-1 can be eliminated from *E. coli* without compromising the reproductive strength of the bacteria. The resulting status of UAG was apparently stable, and could be maintained over >300 generations to isolate B-95.ΔAΔ*fabR*. When 60 of the 95 UAG codons, listed in Supplementary Table 1, had been replaced, we tried to eliminate RF-1. Although RF-1 was successfully eliminated, the resulting strain grew more slowly than BL21(DE3) and B-95.ΔA (Supplementary Fig. 1a), which indicated that the replacement of as many of the 95 UAG codons as possible was necessary to preserve the growth vigor of the bacteria. The introduction of UAG-translating tRNAs into B-95.ΔA immediately changed the status of UAG to a sense codon specifying a natural or non-natural amino acid. Thus, the efficient incorporation of synthetic amino acids and the reproductive strength have made the B-95.ΔA strains superior host organisms for synthesizing recombinant proteins bearing useful modifications. The application of “orthogonal” ribosomes that barely interact with RF-1 is another approach for preventing the use of UAG as a translation stop, and has been successful in enhancing the UAG-mediated incorporation of synthetic amino acids^30^. However, it might be difficult to totally eliminate the ability of RF-1 to interact with the ribosome, while maintaining a functional ribosomal interaction with RF-2, because these two factors share similar ribosome binding manners^31^.

In the absence of RF-1, the in-frame UAG was reportedly translated to glutamine, tyrosine, and tryptophan by endogenous tRNA species^8^. The RF-1 knockout also causes ribosome stalling at UAG, and possibly triggers a number of molecular mechanisms to rescue the ribosomes^17^. The *ssrA* pathway, involving tmRNA, terminates suspended translation and degrades the polypeptides released from the ribosomes^32^. The ribosome-rescue factors YeaJ and ArfA, the latter of which cooperates with RF-2^33^, resolve ribosome stalling without degrading the released polypeptides^34^,^35^. This ribosome-rescuing mechanism can support the expression of protein products from TAG-ending ORFs in the absence of RF-1, and such products have been detected in a *prfA*^−^ background^8^. The status of UAG in B-95.ΔA can be explained on the basis of these known mechanisms. The marginal expression of FLAG-SucB-T7 in B-95.ΔA is explained by the misrecognition of UAG by endogenous tRNAs. More importantly, the expression of FLAG-SucB suggests that the genes still ending with TAG can be expressed in B-95.ΔA, probably through ribosome stalling at UAG and the subsequent release of synthesized proteins. The *ssrA* pathway probably contributes to a reduction in the expression levels of such products, as reduced expression was observed for *ybl129*, *yhbW*, and *frmR*. The introduction of the UAG-translating machinery immediately redefines the status of UAG as a sense codon, and should cause the C-terminal extension of protein products beyond the regular stop sites. The expression of such proteins does not appear to be harmful to B-95.ΔA, in a clear contrast with the previous observation of the adverse effects of the introduction of UAG-translating machinery into the RF-1-free B strain with all of the UAG codons remaining in the genome^8^.

The present results shed new light on the natural processes leading to the unusual codon assignments found in the nuclear codes of certain organisms, which are predominantly stop-codon reassignments as sense codons^36^. Osawa and Jukes have proposed that the meaning of a codon could change safely, when the codon totally disappears from the genome, together with the cognate tRNA, and then reappears with the new meaning conferred by a different tRNA^37^. This process might be accelerated, if the tRNA could be eliminated first, prior to the total disappearance of the codon. Our results support this scenario, particularly for stop codons. With some portion of a stop codon left in the genome, its reassignment as a sense codon would cause the C-terminal extension of the corresponding protein products, through a downstream shift in the position of the translation stop. The extended C-termini might provide an opportunity for new protein functions and traits to evolve^38^. In conclusion, the B-95.ΔA strains facilitate the large-scale production of modified proteins, and also provide a laboratory model for the evolution of the genetic code.

## Methods

### Cell culture

BL21(DE3) was purchased from Novagen. The LB broth (Miller) was purchased from Nacalai Tesque (Japan), the M9 minimal salts premix was purchased from MP Biomedical Japan, and the 2-YT broth was purchased from Invitrogen. The glycerol minimal medium containing l-leucine (GMML)^26^ in our study consisted of M9 minimal medium, 1 mM MgSO_4_, 1% glycerol, 0.3 mM l-leucine, and Kao and Michayluk vitamin solution (Sigma-Aldrich). The GMML auto-induction culture for the HV1 preparation consisted of 0.05% d-glucose, 0.2% d-lactose, 0.5% glycerol, NSP, vitamins, 10 mM *O*-sulfo-l-tyrosine (Watanabe Chemical Industries, Hiroshima, Japan), and carbenicillin (100 μg/ml). The auto-induction medium for the Fab preparation consisted of 2-YT, 1 mM MgSO_4_, 0.05% d-glucose, 0.2% d-lactose, 0.5% glycerol, nitrogen sulfur phosphorus solution (NSP), 1 mM azido-l-phenylalanine (Bachem), and kanamycin (30 μg/ml). The 20-fold concentrated NSP solution (pH6.8) was composed of 0.5 M KH_2_PO_4_ (68 g), 0.5 M Na_2_HPO_4_ (71 g), 1 M NH_4_Cl (53.6 g), and 0.1 M Na_2_SO_4_ (14.2 g) per liter. Anaerobic culturing was performed using Culture set, FX-2 (ISO, Japan). The optical cell densities were measured with an Ultraspec spectrophotometer (GE Healthcare) and Libra S11 Visible and UV spectrophotometers (Biochrom Ltd.).

### Plasmid Construction

All of the plasmids created in the present study are listed in Supplementary Table 10. pMW118 was purchased from Nippon Gene (Japan). To create pBeta, the *lacI^q^*-*tac* promoter^6^, *bet* from DE3, an *rrnB* terminator from pBAD TOPO (Invitrogen), and a *kan* marker were inserted, in this order, between *bla* and the multiple cloning site of pMW118. To derive pRF0q3 from pBeta, the *bla* gene was replaced by a variant of the chloramphenicol acetyltransferase gene (*cat*) with UAG in place of all of the 13 Gln codons^17^, an amber suppressor tRNA^Gln^ *supE3*^17^, and *tetA*. To derive pMINIqY from pBeta, an artificial operon composed of minor tRNA genes, *tetA*, and *lysY* from pLemo (New England Biolabs) was cloned. The tRNA operon consisted of the *tyrT* promoter, tRNA^lle2^-tRNA^Arg3^-tRNA^Pro2^-tRNA^Arg4^_UCG_, *metT-leuW*, tRNA^Arg4^-tRNA^Arg5^, and the *rrnC* terminator. The *bla* gene was removed and the *kan* gene was inactivated by mutation. The temperature-sensitive pMW118 derivative harbored *bla*, in addition to *tetA* and *kan*. pAp15 was constructed from pAp105^17^ by removing TAG at the end of *repZ* and inserting an *rrnC* terminator upstream of the *kan* gene. The *metT-leuW-glnU-glnW-metU-glnV-glnX* operon was cloned in pAp15. *supE44* and *supE3* were cloned in place of *glnX*, to create pAp15-supE44 and pAp15-supE3, respectively. The *hemA-prfA-hemK* operon was cloned into pAp15 to create pAp15-RF1. A SucB variant with a FLAG tag and TAG at the N- and C-termini, respectively, was cloned in pET21b(+) (Novagen), together with the base sequence CAGTTCAAGTTTCACCTGCACTGCAGACCG between the TAG and the start of the T7 tag sequence of the vector, to create the gene encoding the FLAG-SucB-T7 variant. The gene encoding the hirudin variant 1 (HV1) was commercially synthesized, together with the N-terminal 6× His tag and SUMO tag^24^ and designated as SUMO-HV1(63Y). The tyrosine codon at position 63 was mutated to UAG to create SUMO-HV1(63Am). The SUMO-HV1 sequences were each cloned downstream of the T7 promoter of pET21b(+). The artificial operon designated as SfYN3, expressing a UAG-decoding tRNA^Tyr^ variant (Nap3)^39^ and a tyrosyl-tRNA synthetase variant specific to *O*-sulfo-l-tyrosine [SfYRS(D286R)] from *Methanocaldococcus jannaschii*^6^,^23^ was cloned into the pET21b(+) carrying the HV1 gene, to create pET-SUMO-HV1(63Y) and pET-SUMO-HV1(63Am). Then, a *pelB* signal was inserted at the N-termini of the SUMO-HV1, to create pET-pelB-SUMO-HV1(63Y) and pET-pelB-SUMO-HV1(63Am). VH-CH1 and VL-CL, forming the Herceptin Fab fragment, were each cloned with a *pelB* signal in pET26b(+) (Novagen). The Ala codon at position 121 in VHCH1 was changed to UAG to create the pelB-VHCH1(A121X) gene^29^. The *pelB*-VLCL, together with either the *pelB*-VHCH1 or the *pelB*-VHCH1(A121X) was cloned downstream of the T7 promoter in pET26b(+). An artificial operon designated as AzFA1, expressing a UAG-decoding tRNA^Tyr^ variant (pAzPhe1)^39^ and a tyrosyl-tRNA synthetase variant specific to 4-azido-l-phenylalanine [AzFRS(D286R)] from *M. jannaschii*^6^,^26^, was cloned into the pET26b(+) carrying the genes encoding the Fab fragment, to create pET-Fab and pET-Fab(A121X). The 183rd and 201st codons of the heavy chain were changed to TAG to create pET-Fab(3 × Am).

### *E. coli* genome databases

The 95 TAG-ending genes were selected, based on the *E. coli* gene databases, EcoCyc13^40^, EcoGene (with the pseudogene list)^41^, RegulonDB ver. 8^42^, Genobase ver. 8 (http://ecoli.aist-nara.ac.jp/GB8/), WebGeSTer^43^, Profiling of *E. coli* chromosome ver. 4 (http://www.shigen.nig.ac.jp/ecoli/pec/index.jsp), UniProtKB (http://www.uniprot.org/), KEGG (http://www.genome.jp/kegg/kegg2.html), and NCBI (http://www.ncbi.nlm.nih.gov/). The selected genes include the seven essential genes (*murF*, *lolA*, *lpxK*, *hemA*, *hda*, *mreC*, and *coaD*), quasi-essential genes (*sucB*, *ubiF, atpE*, and *fabH*), a few pairs of synthetically quasi-lethal genes (*priBC* and *pgpAC*), some genes involved in the metabolism and catabolism in M9 minimal media with glucose or glycerol, and genes encoding chaperones, sensors, ion channels and transporters, transcriptional regulators, growth-phase regulators, and certain genes necessary for maintaining the fidelity of DNA replication and protein translation. A few other TAG-ending genes were also selected, because they are flanked by or fused to important downstream genes.

### Oligonucleotides used for chromosome modifications

All oligonucleotides were purchased from Sigma Aldrich and used without additional purification. The melting temperatures of the primers used for colony-direct PCR ranged from 58.4 to 62°C, as calculated using the Sigma Aldrich OligoEvaluator (http://www.oligoevaluator.com/). The oligonucleotides with phosphorothioate modifications were designed in such a way that the lengths of the most oligos were 75 bases^18^, with the four 5′-terminal internucleoside linkages being phosphorothioated to resist exonuclease activity^44^. The phosphorothioated oligonucleotides were diluted to 20 μM and then heat-denatured. The salt concentration in the 20 μM solution was moitored as follows. An aliquot of the oligonucleotide solution (3 μl) was mixed with 50 μl of MilliQ-purified water (milliQ water), transferred into an electroporation cuvette (1 mm gap), and electroporated using a BIO-RAD Micro Pulser. When the time constant was <5.5 milliseconds, another aliquot of the oligonucleotide-solution (<3 μl) was mixed with 50 μl of electro-competent cell suspension.

### Oligonucleotide-mediated recombination and colony-direct PCR

BL21(DE3) was transformed with pBeta for chromosomal engineering. To prepare electro-competent cells, cells were cultured in Luria-Bertani (LB) medium containing 30 μg/ml kanamycin and 1 mM isopropyl β-d-1-thiogalactopyranoside (IPTG) in a test tube with vigorous shaking at 37°C, and harvested by centrifugation from 1 ml of culture when the optical density reached 0.5. The cells were then washed twice with ice-cold milliQ water, and suspended in 47 μl of ice-cold milliQ water. One or two of the phosphorothioated oligonucleotides, listed in Supplementary Table 11, in a 3 μl solution were mixed with the cell suspension. The mixture was then chilled on ice for 1 minute and transferred into a 1-mm gap electroporation cuvette, pre-chilled on ice. Transformation was performed using a Bio-Rad Micro Pulser. Efficient transformation required a time constant >5.6 milliseconds. The transformed cells were incubated in 0.5 ml of super optimal broth with catabolic repressor (SOC) in a test tube with vigorous shaking at 37°C for 1.5 hours. The cells were then used for the 2nd cycle of transformation within the same day. After the 2nd cycle, the cells were incubated for 3 hours, diluted 0.4 million times by vortexing, and inoculated on LB agar plates containing kanamycin.

The colonies with the intended base changes were identified by colony-direct PCR. To avoid the accumulation of spontaneous mutations, colonies showing less growth vigor than the neighbors on the agar plate were not picked. To accelerate the screening, eight colonies were mixed in 40 μl of milliQ water and examined at one time, and then the desired clones were identified by a second round of colony-direct PCR. In most of the cases, 256 colonies from eight LB agar plates were examined. Each 10 μl PCR reaction included 5 μl of GoTaq Premix (Promega), 1 μl of primer mixture (0.2 μM final for each), and 4 μl of cell suspension. The PCR program consisted of the following steps: 1) Initial denaturing at 95°C for 2 min 2) Denaturing at 95°C for 30 sec 3) Annealing at 57°C for 30 sec 4) Polymerization at 72°C for 30–40 sec 5) Repeating cycles (2–4) 31 times 6) Graduating polymerization step at 72°C for 2 min

Five-microliter aliquots of PCR samples were analyzed by electrophoresis at 200V using a 2% agarose gel containing ethidium bromide. Since some hit colonies turned out to be a mixture of two different clones, single colony isolation was performed for checking PCR to be performed the next day, prior to starting the next cycle. Thus, 11 genes were engineered per month on average.

### RF1 knockout

To construct B-60.ΔA::Z, the *prfA* gene was knocked-out using a Zeocin resistance gene, as previously described^4^ with a minor modification. To replace the TAG stop codon of *hemA* simultaneously, the left homology arm included the C-terminal 50-nt sequence of *hemA* instead of the N-terminal 50 nucleotides of *prfA*. To construct B-95.ΔA from B-94 pBeta, pBeta was first replaced by pRF0q3. Next, the chromosomal region between *hemA* and *hemK* was removed by an 85-nucleotide oligonucleotide with the sequence shown in Supplementary Table 1, which annealed with only *hemA* and *hemK*, and not *prfA*. TAG at the end of *hemA* was changed to TAA by this recombination. To monitor the disruption of *prfA*, pRF0q3 carrying the SupE3 amber suppressor tRNA^Gln^ gene and the mutant chloramphenicol acetyltransferase (*cat*) gene with all 13 of the glutamine codons replaced by UAG was introduced into B-94. Since this *cat* mutant can be expressed only when RF-1 is absent, RF-1-deficient derivatives of B-94 were easily detected on a Luria-Bertani (LB) agar plate containing 34 μg/ml chloramphenicol (Cm). Only Δ*prfA* Su+ cells were kept. Finally, pRF0q3 was removed by exploiting the temperature-sensitive replication of pMW118, to obtain the plasmid-free B-95.ΔA.

### Whole genome sequencing

Chromosomal DNA was prepared using a Dr. GenTLE yeast genome extraction kit (Takara Bio, Kyoto, Japan). The DNA concentration was determined with a Qubit 2.0 fluorometer (Invitrogen). The genomic DNA was “tagmentated” according to the instructions provided with the Nextera XT DNA sample preparation kit (Illumina). The tagmentated DNA fragments were amplified by PCR and purified with an AMpure XP kit (Beckman Coulter). The prepared libraries were set in MiSeq sequencer ver. 2. The obtained paired-end reads of 2 × 150 bases were mapped, based on the reference genome of BL21(DE3) (NC_012971.2) by using the GENETYX-Genome(64-bit) software. A list of mutations and insertions/deletions was prepared for further analysis.

### 2D-PAGE analysis and MS/MS spot identification

Cells were cultured in LB medium, and harvested in their log phase, when the optical density at 600 nm (OD_600_) was 1.0. The proteomic analysis followed by MS/MS spot identification was commercially performed by Prophoenix (Towa Environment Science Co. Ltd., Japan). Two hundred micrograms of the total cell lysates were analyzed by two-dimensional polyacrylamide gel electrophoresis (2D-PAGE) with a pH range of 3–10 (0.2% Pharmalyte). The first electrophoresis was performed by using CoolPhoreStar IPG-IEF Type-P (Anatech, Japan) and Immobiline DryStrip pH3–10, 18 cm (GE Healthcare) (Step 1: 500V, 0.01 h; Step 2: 3500V, 1.30 h; Step3: 3,500V, 6.00 h). The second electrophoresis was performed by using an Anderson ISO-DALT electrophoresis system (Hoefer) and 20 × 21 cm 9–18% acrylamide concentration gradient gel run for 17 hours at 80 V (constant). Gels were stained with SYPRO Ruby dye, and analyzed by using a Molecular Imager FX (BIO-RAD) and the ImageMaster 2D 5.0 Platinum software (GE Healthcare). Nine protein spots were analyzed by the MS/MS Ions search. They were digested by trypsin, purified with a ZipTip C18 (Millipore), and eluted using a 50% acetonitrile and 0.1% TFA solution. Samples were analyzed by using an Ultraflex TOF/TOF mass spectrometer and an MTP Anchorchip 600/384 target (Bruker Daltonics). The data were analyzed by Mascot (Matrix Science) using the NCBInr database.

### SucB expression

To express the tagged SucB variants, the B-95.ΔAΔ*fabR* and B-94 strains were transformed with pMINIqY, pAp15, pAp15-RF1, pAp15-supE3, and pET-sucB. The transformed cells were cultured in LB medium, containing ampicillin (100 μg/ml) and kanamycin (15 μg/ml) with vigorous shaking at 37°C. Protein expression was induced by the addition of IPTG at a concentration of 1 mM and further incubation for 4 hours. The same volumes of cell cultures adjusted to the same optical density at 600 nm were fractionated on a NuPAGE gel (Life Technologies) and analyzed by western blotting using peroxidase-conjugated antibodies against the FLAG-tag and the T7-tag (from Sigma-Aldrich and Novagen, respectively).

### Expression and purification of recombinant hirudin

To produce the 6× His-SUMO-tagged hirudin variant 1 (HV1) in the cytoplasm, BL21(DE3) and B-95.ΔAΔ*fabR* were transformed with either pET-SUMO-HV1(63Y) or pET-SUMO-HV1(63Am). The transformed cells were incubated in the GMML medium containing 0.4% glucose, *O*-sulfo-l-tyrosine (10 mM), and carbenicillin (100 μg/ml) at 37°C, until the OD_600_ reached 0.4. IPTG was then added at a final concentration of 0.5 mM, and the cells were incubated at 25°C for 16 h. The harvested cells were lysed by sonication. The 6× His-SUMO-tagged HV1 was purified by chromatography on a HisTrap column using an AKTA Explorer, with a buffer containing 20 mM sodium-phosphate (pH7.4), 500 mM NaCl, and 10 or 500 mM imidazole. SUMO protease was added to the eluate, to cleave the tag. The incubation with the enzyme was performed at 4°C overnight, simultaneously with the dialysis to reduce the imidazole concentration to 10 mM, using a Spectra/Por Dialysis Membrane MWCO 1,000 (Spectrum Labs). The N-terminal tag was removed with a His SpinTrap column (GE Healthcare), and the eluate containing HV1 was then dialyzed against PBS. The yields of HV1 were determined using a BCA Protein Assay kit (Reducing Agent Compatible) (Thermo Scientific). To synthesize the 6× His-SUMO-tagged HV1 in the secreted form, BL21(DE3) and B-95.ΔAΔ*fabR* were transformed with either pET-pelB-SUMO-HV1(63Y) or pET-pelB-SUMO-HV1(63Am). The transformed cells were inoculated in the auto-induction medium based on GMML and cultured at 30°C for 40 h. The products were purified from the growth medium by chromatography on a Ni-Sepharose column, and then purified by the aforementioned steps.

### Matrix-assisted laser desorption ionization time-of-flight (MALDI-TOF) mass spectrometric analysis of recombinant hirudin

HV1 was reduced by an incubation in Bond-Breaker TCEP solution (neutral pH) (Thermo Scientific) with TECP at a final concentration of 50 mM at 70°C for 10 min, and then desalted by using a SPE C-TIP(C18) column (Nikkyo Technos, Co., Ltd., Japan), followed by elution in 80% acetonitrile and 0.5% TFA. The obtained samples were analyzed by MALDI-TOF MS in a TOF/TOF 5800 system (AB SCIEX) in the positive mode with sinapinic acid (Tokyo chemical industry Co. Ltd., Japan) or 2′, 6′-dihydroxyacetophenone (Sigma-Aldrich) as the matrix.

### Anti-thrombin activity assay

The anti-thrombin activity of HV1 was examined by the fluorogenic enzyme assay described previously^23^. Human α-thrombin (0.2 NIH unit) (Haematologic Technologies) and HV1 (at a final concentration of 7.5 nM) were mixed in a buffer containing 50 mM Tris-HCl (pH 8.0), 154mM NaCl, and 0.2% PEG6000, and then pre-incubated for 3 min, prior to the addition of the fluorogenic thrombin substrate Boc-Asp(OBzl)-Pro-Arg-MCA (50 μM final) (Peptide Institute, Japan). The reaction volume was 1 ml. The commercially available recombinant hirudin from yeast (H0393-100UN, Sigma-Aldrich) was used as a control for the assay. The release of the fluorescent dye by thrombin was monitored in a 96-well plate at room temperature using an ARVO X3 spectrometer (Perkin-Elmer) with the excitation at 355 nm and emission monitored at 460 nm.

### Expression, purification, and modification of Fab

BL21(DE3) and B-95.ΔA were transformed with pET-Fab and pET-Fab(Am121). The transformed cells were cultured in 5 ml of the auto-induction medium, with vigorous shaking at 25°C for 22 hours. The harvested cells were lysed in a BugBuster Master Mix solution (Novagen) using 500 μl solution per 100 mg cells. The lysate (400 μl) was mixed with Protein G Mag Sepharose Xtra magnetic beads (100 μl) (GE Healthcare), as described previously^45^. Fab fragments were eluted in an acidic condition, and the eluate (100 μl) was then neutralized by the buffer included in the Ab buffer kit (20 μl) (GE Healthcare). The yields of Fab fragments were determined based on the absorption at 280 nm, using a NanoDrop 2000c spectrometer (Thermo Scientific). The purified Fab was fractionated on a NuPAGE gel and detected using the SimplyBlue SafeStain (Life Technologies). The Rhodamine-triarylphosphine (RTP) conjugate and the FITC-triarylphosphine (FTP) conjugate were commercially synthesized by Shinsei Chemical Co. Ltd. (Osaka, Japan). The RTP and FTP were dissolved in dimethyl sulfoxide at the concentrations of 4 mg/ml and 5 mM, respectively. The solution containing the purified Fab was mixed with one-twentieth volume of RTP or FTP, and then incubated at 37°C for 30 minutes. The labeled Fab fragments were fractionated on a NuPAGE gel and then visualized using an LAS4010 spectrometer (GE Healthcare).

### Fab binding assay

The affinity of a Fab fragment to the ERBB2 extracellular domain was determined using a Biacore 3000 and a Human Fab Capture kit with the HBS-P running buffer (GE Healthcare). A Fab fragment (5 μg/ml) was loaded on a CM5 sensor chip coupled with the Fab Binder, included in the kit, at a 10 μl/min flow rate for 3 min. Various concentrations of the ERBB2 extracellular domain (0.5 nM, 1 nM, 2 nM, 4 nM, and 8 nM) were then loaded at a 30 μl/min flow rate for 3 min, followed by the HBS-P running buffer for 3 min, to analyze the interaction between the Fab and ERBB2.

## Author Contributions

T.M. and K.S. designed the research and wrote the paper. T.M. developed and analyzed bacterial strains. H.H. performed a preliminary study. T.M., K.O., A.H. and M.T. performed recombinant protein experiments. A.Y. performed MS analyses. T.M., H.H., K.O., M.T., A.Y., A.H., S.Y. and K.S. interpreted the data.

## Additional Information

**How to cite this article**: Mukai, T. *et al.* Highly reproductive *Escherichia coli* cells with no specific assignment to the UAG codon. *Sci. Rep.* 5, 9699; DOI:10.1038/srep09699 (2015).

## Supplementary Material

Supplementary Information Supplementary Information

## Figures and Tables

**Table 1:** Off-target mutations in the B-95.ΔA genome. The repeated DNA sequences, including rDNA, insertion elements, and *rhs* genes, were not fully analyzed

Annotation	Position	Type	Reference	Sequenced
*cdaR(L52L)*	185461	Substitution	G	A
upstream of *sulA*	1026026	Substitution	T	C
terminator of *pspF*	1353260	Substitution	G	A
*dcp(ΔA)*	1575919	Deletion	T	
*yfbS(L39L)*	2298490	Substitution	A	G
*hemX(P389PAP)*	3878170-1	Insertion		GGTGCA
*tolQ(L130L)*	734203	Substitution	C	T
*minE(Q67P)*	1212505	Substitution	T	G
*yjhB(ΔT)*	4415207	Deletion	T	

**Table 2:** Doubling times and maximal cell densities of B-94, B-95.ΔA, and B-95.ΔAΔ*fabR* in minimal and rich media. The cells were incubated in a test tube with vigorous shaking. The average values from three independent experiments and the standard deviations are shown

Strain	Medium	Temperature	Doubling time (h)	Max OD_600_
B-94	M9 glucose	37°C	1.52 ± 0.05	2.02 ± 0.14
B-95.ΔA	M9 glucose	37°C	2.13 ± 0.03	1.52 ± 0.04
B-95.ΔAΔ*fabR*	M9 glucose	37°C	1.78 ± 0.01	2.06 ± 0.14
B-94	LB	16°C	3.14 ± 0.11	4.96 ± 0.08
B-95.ΔA	LB	16°C	3.66 ± 0.04	4.09 ± 0.07
B-95.ΔAΔ*fabR*	LB	16°C	3.38 ± 0.06	5.10 ± 0.02
