# Supplementary material for: Highly reproductive Escherichia coli cells with no specific assignment to the UAG codon
Source: Sci Rep. 2015 May 18;5:9699. doi: 10.1038/srep09699 (PMC4434889; doi:10.1038/srep09699)
Supplement: Supplementary Information [file srep09699-s1.pdf]

SUPPLEMENTARY INFORMATION

Highly reproductive *Escherichia coli* cells with no specific assignment to the UAG codon

Takahito Mukai, Hiroko Hoshi, Kazumasa Ohtake, Mihoko Takahashi, Atsushi Yamaguchi, Akiko Hayashi, Shigeyuki Yokoyama & Kensaku Sakamoto*

*Author for correspondence. E-mail: kensaku.sakamoto@riken.jp;

Phone: +81-45-503-9459.

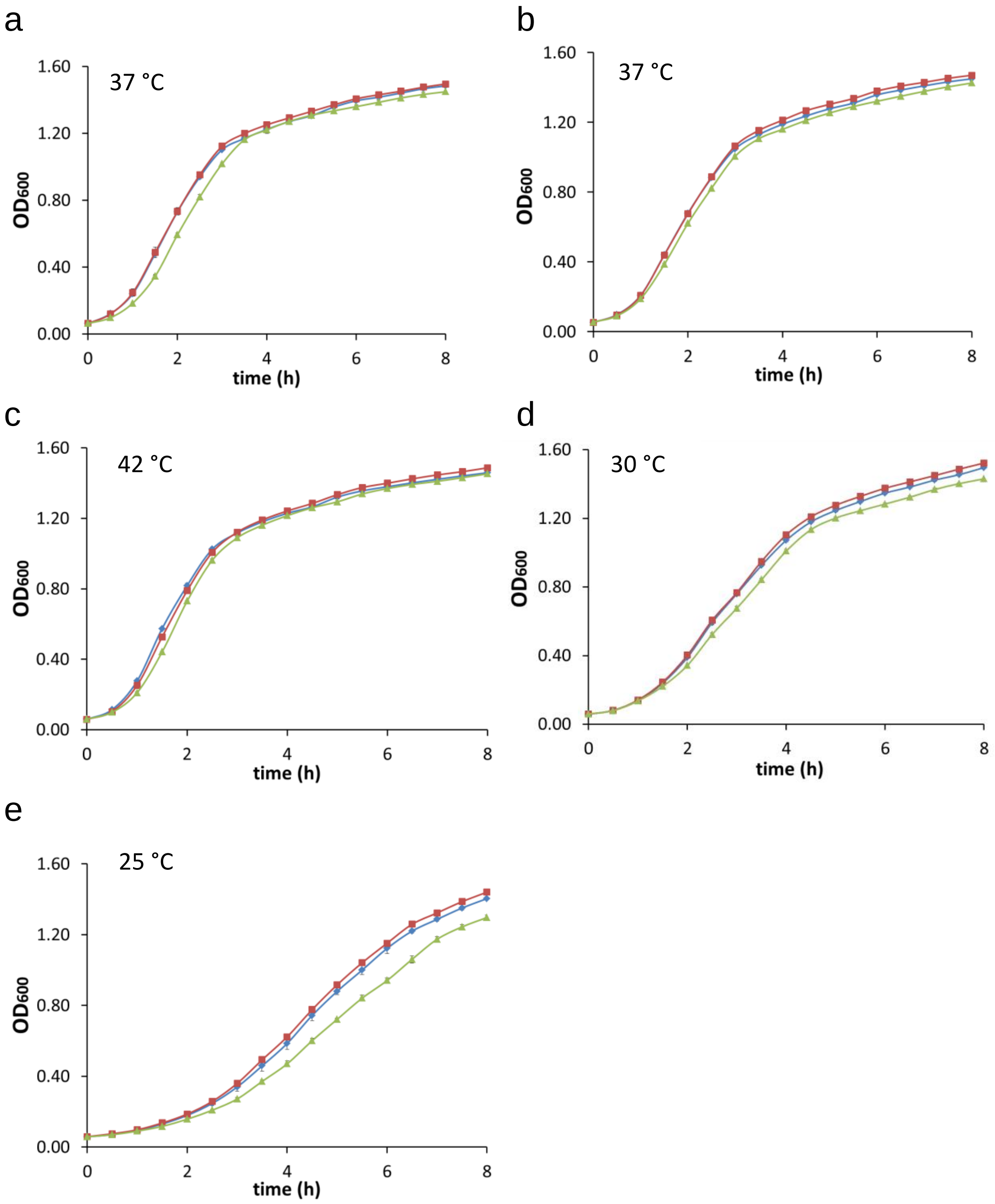

Supplementary Figure 1 | Growth profiles of BL21(DE3), B-59, B-60.ΔA::Z, B-94, and B-95.ΔA in rich medium. The data were obtained from three independent experiments. (a) Growth curves of BL21(DE3) (blue), B-59 (red), and B-60.ΔA::Z (green) at 37 °C. (b—e) Growth curves of BL21(DE3) (blue), B-94 (red), and B-95.ΔA (green) at 37 °C, 42 °C, 30 °C, and 25 °C, as indicated.

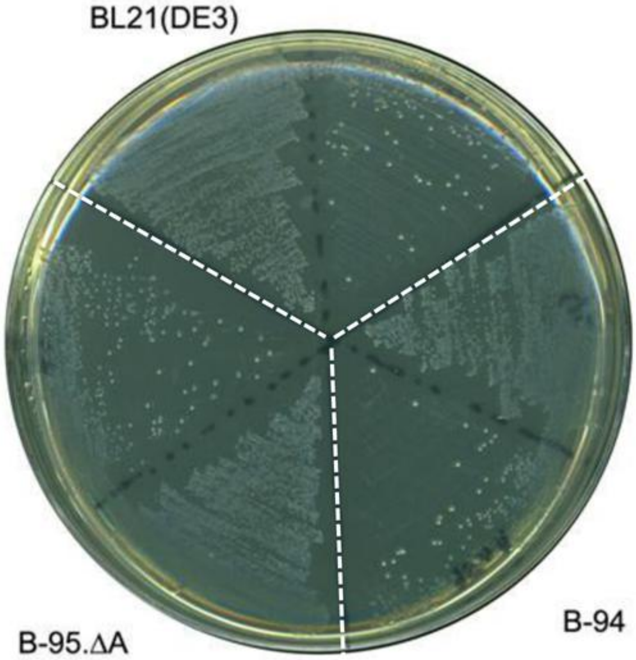

Supplementary Figure 2 | Anaerobic growth of BL21(DE3), B-94, and B-95.ΔA. These strains were inoculated on an LB agar plate and incubated at 37°C for 2 days in an anaerobic culture pack. Oxygen was fully removed, as confirmed by an indicator tablet.

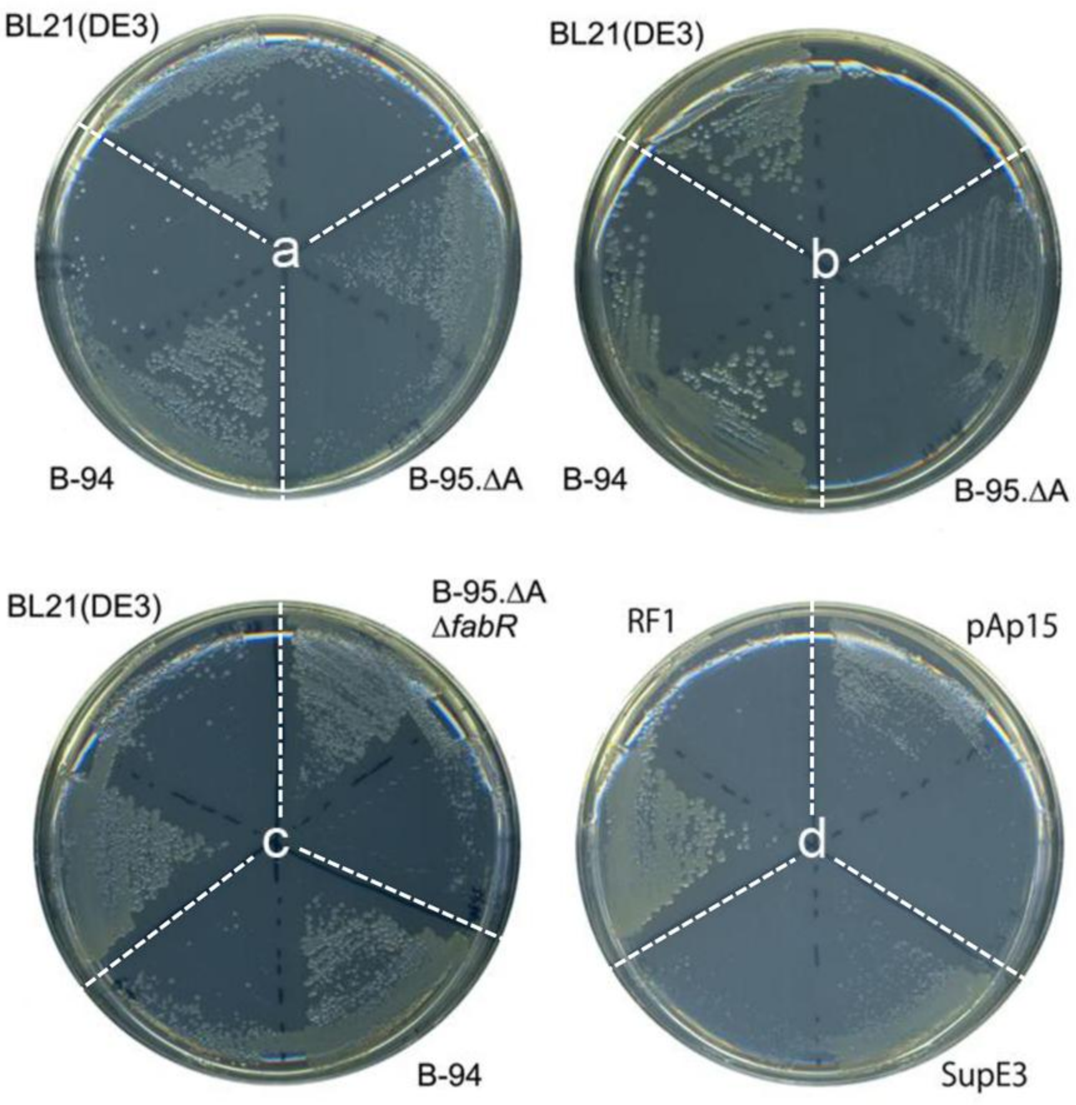

Supplementary Figure 3 | Growth of BL21(DE3), B-94, B-95.ΔA, and B-95.ΔAΔ*fabR* in M9 minimal media at 37°C. The inoculated cells had been pre-cultured in the same media at 37°C. (**a**) B-95.ΔA, B-94, and BL21(DE3) were incubated on an M9 glucose plate for 1 day. (**b**) B-95.ΔA, B-94, and BL21(DE3) were incubated on an M9 glycerol plate for 2 days. (**c**) B-95.ΔAΔ*fabR*, B-94, and BL21(DE3) were incubated on an M9 glycerol plate for 2 days. (**d**) B-95.ΔAΔ*fabR* cells transformed with pAp15, pAp15-RF1, and pAp15-supE3 were incubated on a GMML plate containing vitamins and kanamycin (15 μg/ml) for 2 days.

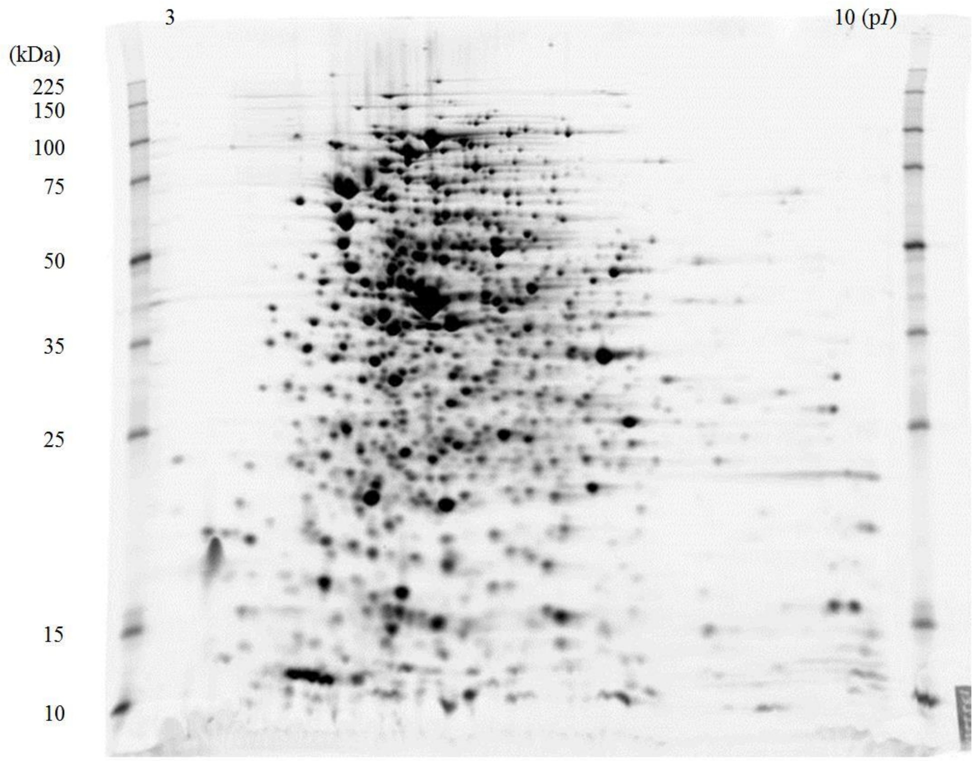

proteins: 200 µg BL21(DE3) SYPRO Ruby dye

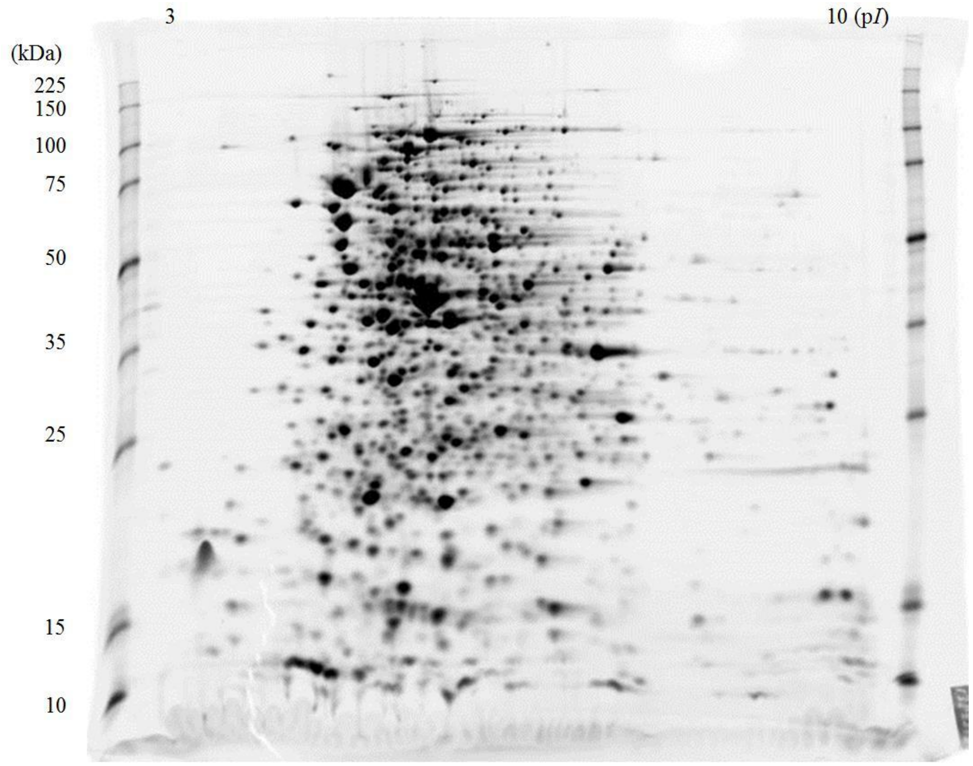

proteins: 200 µg B-94 SYPRO Ruby dye

(Part of Supplementary Figure 4)

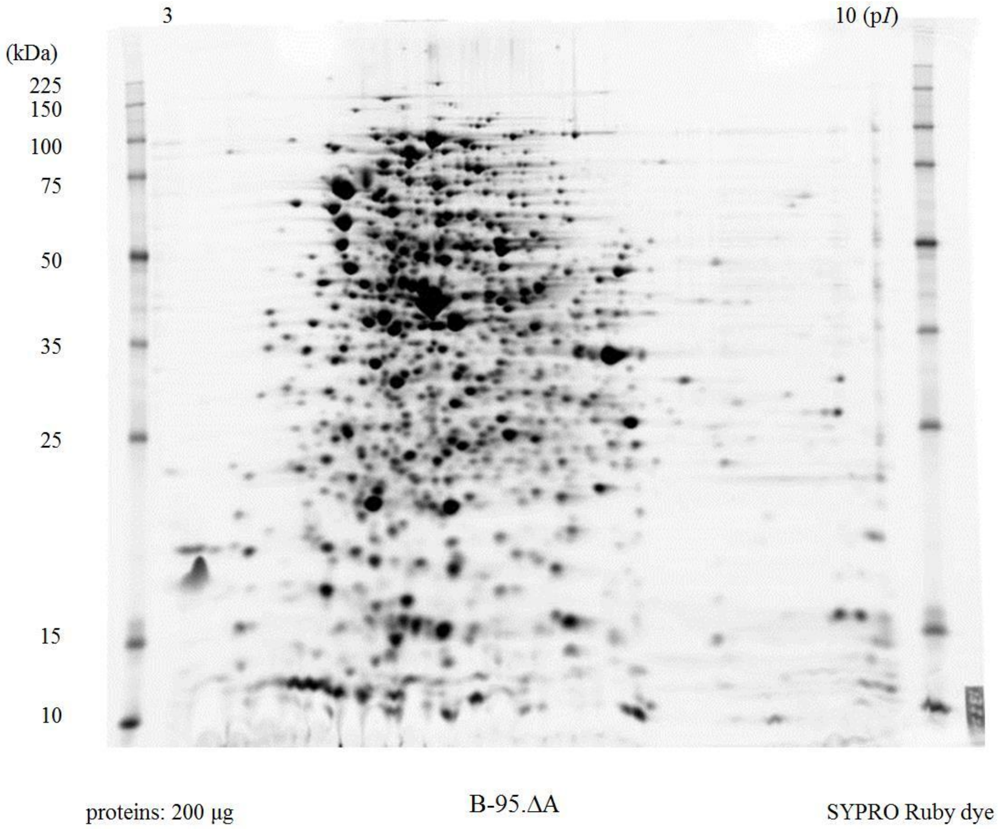

Supplementary Figure 4 | 2D-PAGE analyses of the total proteins extracted from BL21(DE3), B-94, and B-95.ΔA at the exponential growth phase in LB medium.

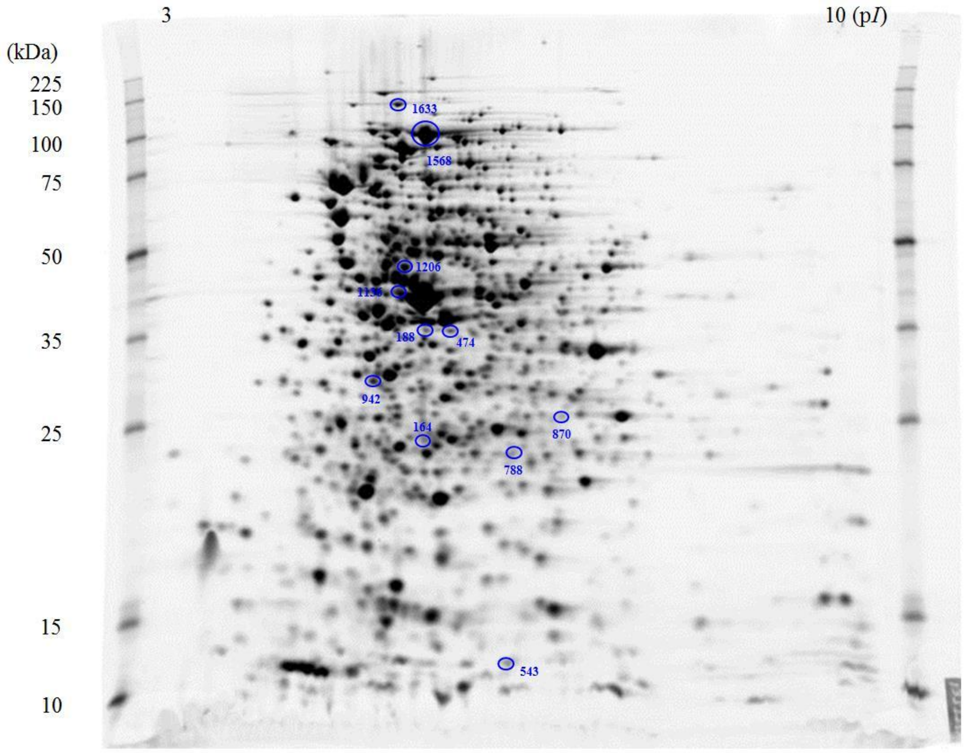

proteins: 200 µg BL21(DE3) SYPRO Ruby dye

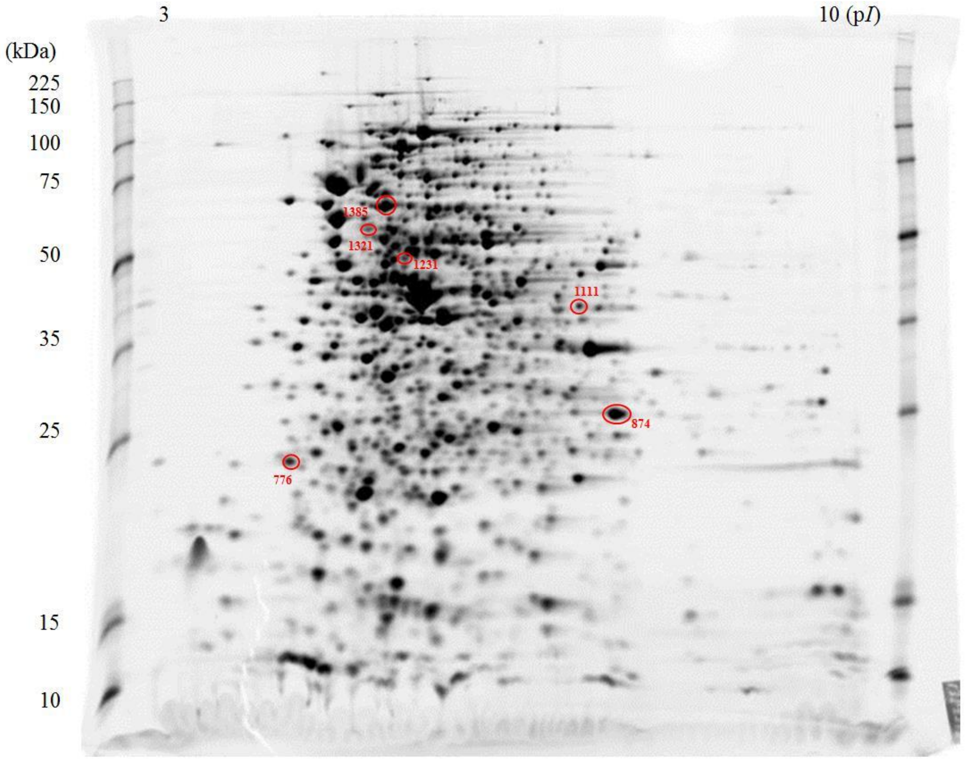

proteins: 200 µg B-94 SYPRO Ruby dye

(Part of Supplementary Figure 5)

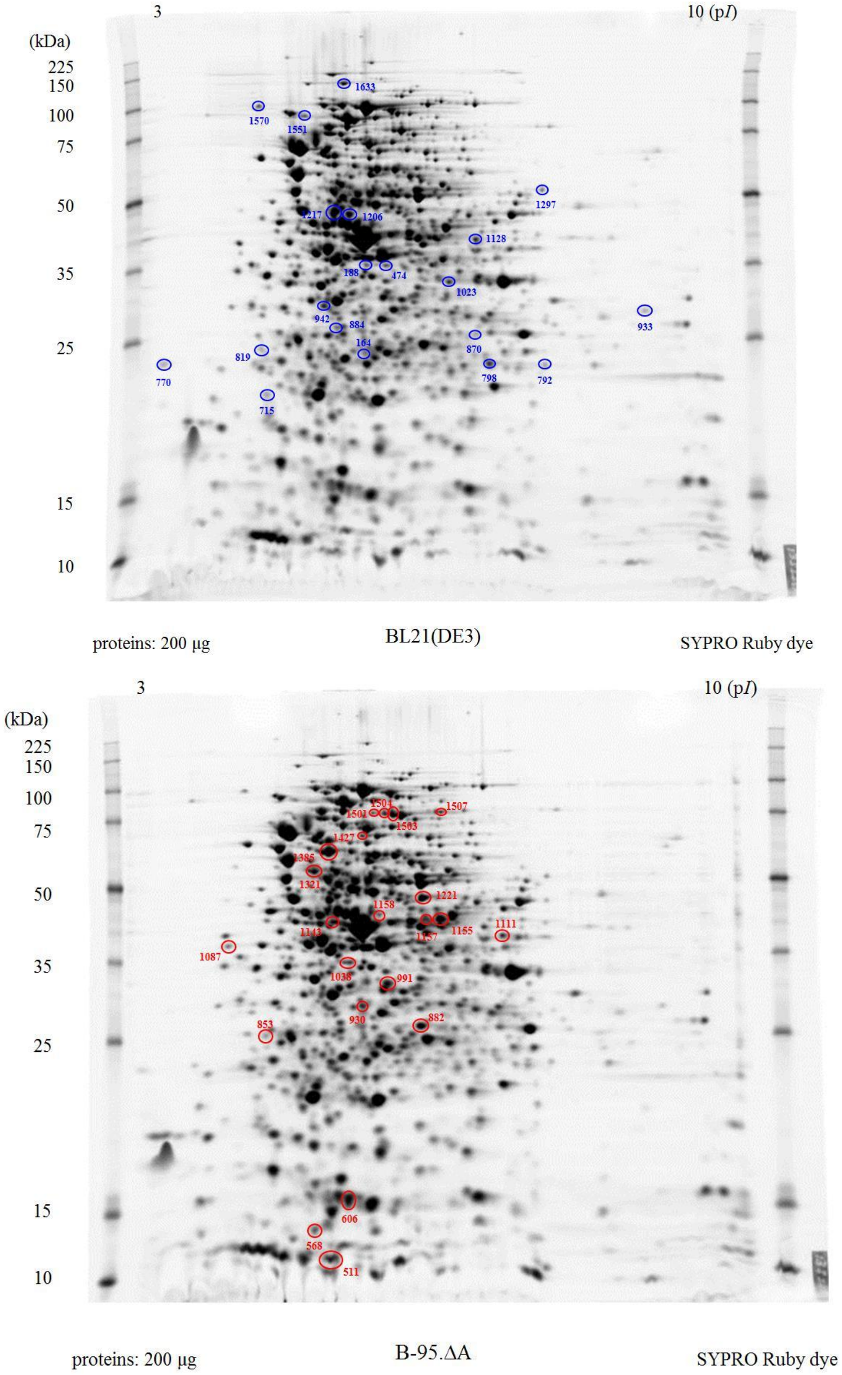

Supplementary Figure 5 | Annotations of the protein spots from the 2D-PAGE analyses shown in Supplementary Figure 4. Protein spots showing significant changes in the intensities are circled and numbered after comparing the spot patterns between BL21(DE3) and B-94 and between BL21(DE3) and B-95.ΔA.

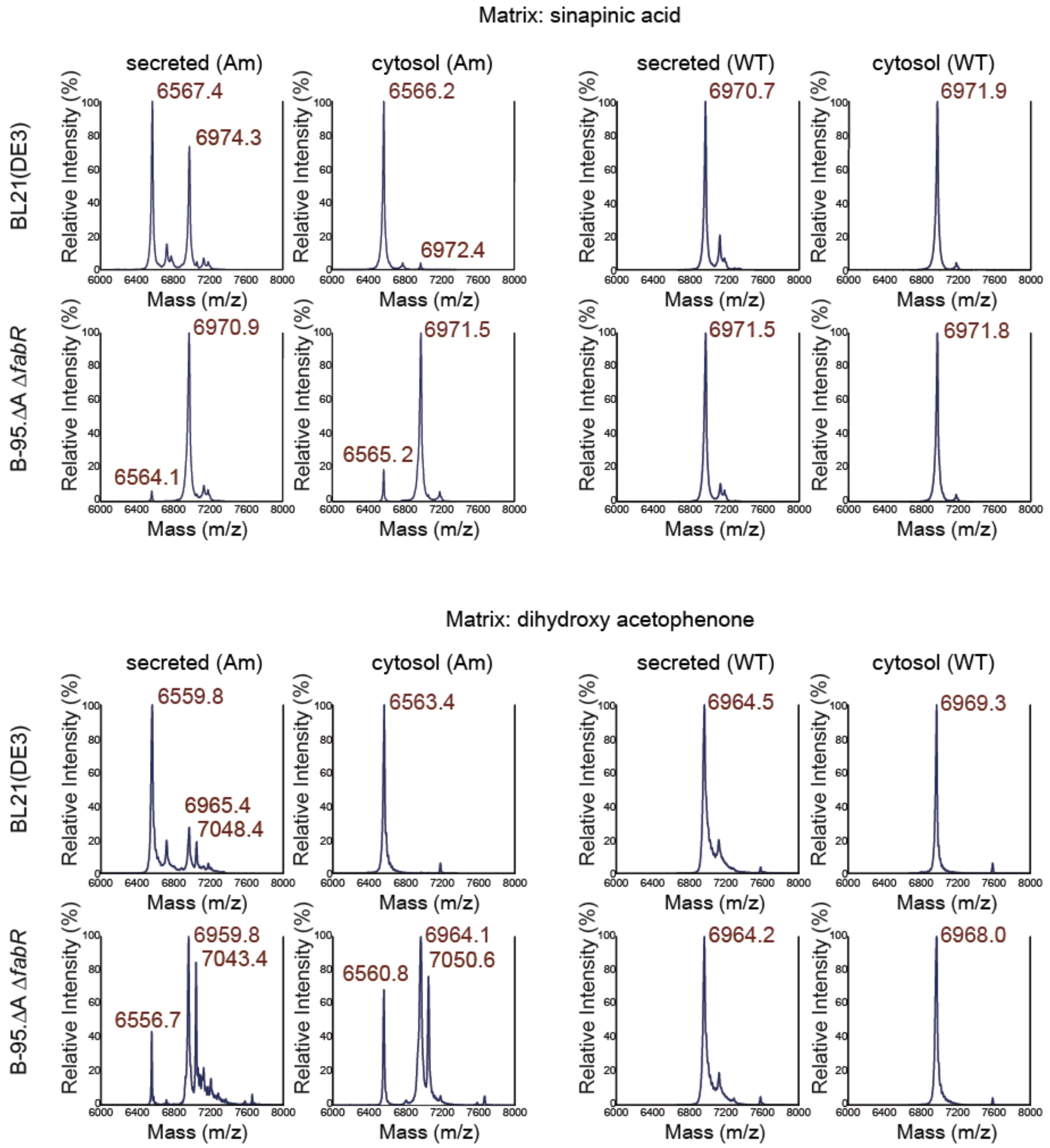

Supplementary Figure 6 | MALDI-TOF spectra of the recombinant HV1. “Am” and “WT” indicate that HV1 was expressed from a gene with UAG at position 63 and another with no in-frame stop codon, respectively. The calculated masses (m/z) of the full-length HV1 with and without *O*-sulfation are 7050.5 and 6970.5, respectively, for $[M+H]^+$. The calculated mass (m/z) for the truncated HV1 is 6566.1 for $[M+H]^+$. The sulfotyrosine was deacylated with the sinapinic acid matrix. The second matrix prevented the deacylation to some extent.

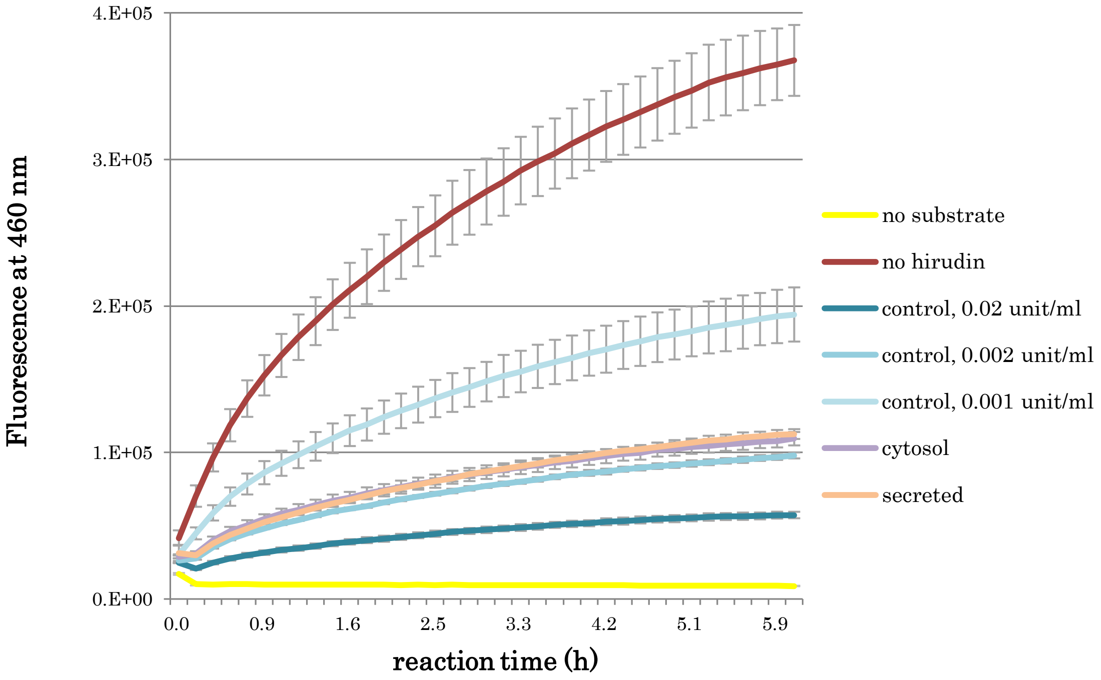

Supplementary Figure 7 | Anti-thrombin activity of the recombinant sulfo-hirudin from B-95.ΔAΔ*fabR*, either expressed in the cytoplasm or secreted into the periplasm. Fluorescent intensity was measured at ten-minute intervals for about six hours. The activity of the control hirudin, serially diluted from 0.001, 0.002, to 0.02 anti-thrombin unit (ATU) / ml, is shown for comparison. The standard deviations are from three independent experiments.

Supplementary Table 1 | All 273 of the genes ending with TAG in *E. coli* B strains. The column "strain B specific" indicates whether the listed genes end with TAG specifically in *E. coli* B strains (or commonly in B and K-12 strains). The column "engineered in" indicates whether TAG at the end of the gene is replaced in B-95.ΔA, in both B-95.ΔA and B-60.ΔA::Z, or not replaced. The genes are categorized, based on their involvement in 1) translation & replication, 2) lipids, envelope, cell division, 3) energy & carbohydrates, 4) amino acids, nucleotides, cofactors, 5) inorganic molecules & others, 6) stress response & detoxification, 7) DNA/RNA/protein repair & degradation, 8) growth phase & starvation response, 9) virulence & intercellular communication, 10) predicted proteins & function unknown, and 11) transposons & prophages.

gene ID	gene name	strain B specific	engineered in	annotation	category
ECD_03637	*rbsB*	no		D-Ribose transporter subunit	4
ECD_03639	*rbsR*	no		Ribose operon repressor	4
ECD_03645	*ilvL*	no		*IlvGMEDA* operon leader peptide	4
ECD_03650	*ilvA*	no	B-95.ΔA	Threonine deaminase	4
ECD_03663	*wzzE*	no	Both	Lipopolysaccharide biosynthesis	2
ECD_03675	*aslB*	no		Arylsulfatase-activating protein	5
ECD_03677	*hemY*	no	Both	Protoporphyrinogen oxidase	4
ECD_03693	*ybl179*	yes		Function unknown	10
ECD_03697	*yigG*	no		Inner membrane protein	9
ECD_03701	*recQ*	no	Both	ATP-dependent DNA helicase	1
ECD_03725	*rmuC(yigN)*	no	Both	DNA recombination	1
ECD_03732	*tatD*	no	B-95.ΔA	Mg-dependent cytoplasmic DNase	7
ECD_03757	*yihL*	no		GntR family transcriptional regulator	3
ECD_03769	*yihW*	no	Both	Glycerol-3-phosphate regulon repressor	3
ECD_03773	*yiiD*	no		Putative thioesterase	10
ECD_03799	*cpxP*	no	Both	Periplasmic protein combats stress	9
ECD_03838	*frwD*	no		Enzyme IIB component of PTS	3
ECD_03881	*zraR*	no		Responsive to Zn(2+) and Pb(2+)	6
ECBD_3978	*yjbS*	no		Function unknown	10
ECD_03999	*yjdK(ghoS)*	no		Antitoxin of GhoTS toxin-antitoxin pair	8
B21_03961	*yjdO(ghoT)*	no		Toxin of GhoTS toxin-antitoxin pair	8
ECD_04065	*ulaF(sgaE)*	no	Both	L-ribulose-5-phosphate 4-epimerase	4
ECD_04066	*yjfY*	no		YhcN family	10
ECD_04068	*priB*	no	Both	Primosomal replication	1
ECD_04078	*ytfB*	no		OapA family protein	2

ECD_04083	*ytfG(qorB)*	no	B-95.ΔA	Quinone oxidoreductase B	3
ECD_04087	*ytfJ(ecfJ)*	yes	B-95.ΔA	Periplasmic	9
ECD_04090	*msrA*	no	Both	Peptide methionine sulfoxide reductase	7
ECD_04092	*ytfN(tamB)*	no		Translocation and assembly module	9
ECD_04130	*idnR*	no		*idn* operon activator	3
ECD_04153	*fecE*	no		Ferric citrate ATP-binding	5
ECD_04178	*nanC(yjhA)*	no		*N*-acetylneuraminic acid import	3
ECD_04205	*yjiN*	no		Zinc-type alcohol dehydrogenase-like	10
ECD_04207	*yjiPQ*	no		Transposase family	11
ECD_04212	*mcrB*	no		McrBC restriction endonuclease	6
ECD_04242	*yjjQ*	no		Methylglyoxal-resistance	6
ECD_04252	*ytjA*	no		Widespread in Bacteria	10
ECD_04254	*yjjV*	no		Predicted DNase	7
ECD_04270	*yjjX*	yes	Both	Inosine/xanthosine triphosphatase	4
ECD_04275	*creC*	no	Both	Sensory histidine kinase	8
ECD_00050	*kefF(yabF)*	no		K+ efflux system ancillary protein for KefC	5
ECD_00067	*yabI*	no		DedA family	2
ECD_00068	*thiQ(yabJ)*	no	B-95.ΔA	ABC transporter ATPase for thiamine	4
ECD_00087	*murF*	no	Both	UDP-MurNAc-pentapeptide synthetase	2
ECD_00100	*mutT*	no	Both	8-oxo-dGTP diphosphatase	7
ECD_00112	*pdhR*	no	B-95.ΔA	Pyruvate dehydrogenase operon repressor	3
ECD_00145	*sfsA*	no		Sugar fermentation stimulation	3
ECD_00157	*btuF(yadT)*	no	Both	Vitamin B12 transporter subunit	4
ECD_00161	*cdaR*	no		Carbohydrate diacid regulator	3
ECD_00163	*yaeI*	no		Metallophosphoesterase	10
ECD_00213	*yhhI-1(ybl10)*	no		Predicted transposase	11
ECD_00222	*yafL*	no		Probable endopeptidase	2
ECD_00224	*fhiA*	yes		Lateral flagellar assembly protein fragment	9
ECD_00251	*matC(yagY)*	no		*E. coli* common pilus chaperone	9
ECD_00257	*ykgA*	yes		AraC family	10
ECD_00281	*yahL*	no		Function unknown	10
ECD_00282	*yahM*	no		Function unknown	10
ECD_00289	*prpE*	no		Propionyl-CoA synthetase	3
ECD_00292	*cynR*	no		Activator for the *cyn* operon	5
ECD_00311	*frmR*	no		Negative regulator of the *frmRAB* operon	6

ECD_00344	*araJ*	no		MFS transporter	10
ECD_00366	*pgpA*	no	Both	Phosphatidylglycerophosphatase A	2
ECD_00372	*yajL(thiJ)*	no	Both	Chaperone for sulfenylated thiol proteins	7
ECD_00373	*panE(apbA)*	no	Both	2-dehydropantoate 2-reductase	4
ECD_00377	*ybl12*	yes		GCN5-related N-acetyltransferase	10
ECD_00391	*lon*	no		Lon protease (lacking promoter)	7
ECD_00400	*mdlA*	no		ABC exporter permease-ATPase	6
ECD_00407	*ybaA*	no		DUF1428 family	10
ECD_00412	*ybaJ(tomB)*	no		Hha toxicity modulator	9
ECD_00418	*priC*	no	Both	Primosomal replication	1
ECD_00427	*aes*	no		Acetyl esterase	3
ECD_00471	*ybcF(arcC)*	no		Carbamate kinase	5
B21_00492	*ybl14*	no		Excisionase	11
ECD_00495	*ybcL*	no		Raf kinase inhibitor RKIP homolog	11
ECD_00508	*rzpD(ybcT)*	no		Rz endopeptidase	11
ECD_00547	*hokE*	yes		Hok/gef cell toxic protein	11
ECD_00568	*ybdL*	no		Methionine aminotransferase	4
ECD_00602	*rlpA*	no	B-95.ΔA	Septal ring protein	2
ECD_00630	*ubiF*	no	Both	Ubiquinone biosynthesis hydroxylase	3
ECD_00666	*ybfD*	no		Putative DNA ligase	11
ECD_00674	*nei*	no	Both	Endonuclease 8	7
ECD_00675	*abrB*	no	Both	Regulator of *aidB* expression	6
ECD_00686	*sucB*	no	Both	Pyruvate dehydrogenase (E2 component)	3
ECD_10027	*Q*	yes		Late gene regulator	11
ECD_10031	*Rz*	yes		Cell lysis protein	11
ECD_00745	*bioD*	no	B-95.ΔA	Dethiobiotin synthase	4
ECD_00752	*moaE*	no	Both	Molybdopterin synthase subunit	4
ECD_00754	*ybhM*	no		Inner membrane protein	10
ECD_00760	*ybhS*	no		Predicted ABC transporter permease	10
ECD_00763	*ybiH*	no		TetR family	10
ECD_00785	*ybiR*	no		Putative transporter	10
ECD_00799	*gsiD(yliD)*	no	B-95.ΔA	Glutathione ABC transporter permease	5
ECD_00804	*yliI*	no		Soluble aldose sugar dehydrogenase	3
ECD_00813	*ybjK(rcdA)*	no	B-95.ΔA	Regulator of *csgD*	9
ECD_00814	*ybl27*	yes		Function unknown	11

ECD_00818	*ybl30*	yes		Function unknown	11
ECD_00830	*ybl34*	yes		Function unknown	11
ECD_00832	*ybl36*	yes		Capsid portal protein	11
ECD_00843	*ybl45*	yes		Baseplate J family protein	11
ECD_00847	*tfaE*	no		Tail fiber asembly	11
ECD_00857	*rimK*	no	B-95.ΔA	Ribosomal protein S6 modification	1
ECD_00872	*amiD(ybjR)*	no		*N*-acetylmuramoyl-l-alanine amidase	2
ECD_00885	*cspD*	no	Both	Inhibitor of DNA replication	8
ECD_00895	*lolA*	no	Both	Outer membrane lipocarrier protein	2
ECD_00917	*ycaI*	no	Both	Upstream of *msbA*	10
ECD_00919	*lpxK*	no	Both	Tetraacyldisaccharide 4'-kinase	2
ECD_00930	*ycbK*	no		Peptidase homolog	10
ECD_00951	*ycbX*	no	Both	6-*N*-hydroxylaminopurine resistance	7
ECD_01008	*ymdF*	no		Function unknown	10
ECD_01028	*insF-1*	no		*IS3* transposase B	11
ECD_01054	*yceO*	no		Expressed protein	9
ECD_01083	*yceF*	no	Both	Maf-like protein	7
ECD_01086	*plsX*	no	Both	Fatty acid/phospholipid synthesis	2
ECD_01087	*fabH*	no	Both	Beta-Ketoacyl-ACP synthase III	2
ECD_01092	*pabC*	no	Both	Aminodeoxychorismate lyase	4
ECD_01136	*ycgX*	no	B-95.ΔA	DUF1398 family protein	11
ECD_01142	*ymgC*	no		BluRF and RpoS regulons	6
ECD_01171	*ycgY*	no		Function unknown	10
ECD_01185	*hemA*	no	Both	Glutamyl-tRNA reductase	4
ECD_01224	*kch*	no		Potassium (K^+) channel	5
ECD_01270	*sapB*	no		Peptide transport system permease	9
ECD_01280	*pspF*	no		Psp operon transcriptional activator	6
ECD_01294	*ycjU*	no	B-95.ΔA	Beta-phosphoglucomutase	3
ECD_01302	*ycjG*	no		L-Ala-D/L-Glu epimerase	2
ECD_01306	*ycjZ*	no	B-95.ΔA	Peptide glycan recycling regulator	2
ECD_01322	*intR*	no		Integrase	11
ECD_01331	*sieB*	no		Superinfection exclusion protein	11
ECD_01360	*insF-2*	yes		*IS3* transposase B	11
ECD_01388	*ydcL*	no	B-95.ΔA	Hypothetical lipoprotein	10
ECD_01414	*rhsE*	no		RhsE element core protein	10
ECD_01428	*yddJ*	no		Putative Psedogene	10

ECD_01441	*ddpF(yddO)*	no		d,d-dipeptide permease system	10
ECD_01448	*yddV(dosC)*	no	Both	Diguanylate cyclase	8
ECD_01485	*ydeA(sotB)*	no		Arabinose efflux transporter	3
ECD_01490	*marA*	no		Multiple antibiotic resistance	6
ECD_01491	*marB*	no		Multiple antibiotic resistance	6
ECD_01525	*insF-4*	yes		*IS3* transposase B	11
ECD_01553	*speG*	no	B-95.ΔA	Spermidine acetyltransferase	5
ECD_01560	*dmsD(ynfI)*	no	Both	Tat proofreading chaperone	3
ECD_01619	*ydhM(nemR)*	no		*nemRA-gloA* operon repressor	6
ECD_01623	*lhr*	no		Probable ATP-dependent helicase	10
ECD_01647	*ynhG*	no		Murein l,d-transpeptidase	2
ECD_01672	*ydiA(ppsR)*	no	Both	PEP synthase regulation	3
ECD_01674	*ydiE*	no		Belongs to the Fur regulon	10
ECD_01677	*nlpC*	no	B-95.ΔA	Putative PG hydrolase	2
ECD_01680	*btuC*	no	B-95.ΔA	Vitamin B12 ABC transporter permease	4
ECD_01719	*ydjX*	no		TVP38/TMEM64 family	10
ECD_01728	*nudG*	no	Both	CTP pyrophosphohydrolase	7
ECD_01757	*yoaI*	no		Function unknown	10
ECD_01758	*yeaL*	no		UPF0756 family	10
ECD_01773	*yeaX*	no		Putative oxygenase reductase component	10
ECD_01814	*yobB*	no		Function unknown	10
ECD_01840	*yecN*	no		MAPEG family	10
ECD_01865	*otsA*	no	Both	Trehalose phosphate synthase	6
ECD_01897	*cbl*	no		Activator for *ssuEADCB* and *tauABCD*	5
ECD_01920	*hisL*	no		*his* operon leader peptide	4
ECD_01949	*wcaM*	no		Colanic acid biosynthesis protein	9
ECD_01950	*wcaL*	no		Glycosyl transferase	9
ECD_01963	*wcaC*	no		Glycosyl transferase	9
ECD_01970	*asmA*	no	B-95.ΔA	Outer membrane protein assembly	2
ECD_01984	*baeR*	no	Both	Bacterial Adaptive Response	6
ECD_02028	*yegV*	no		Predicted sugar/nucleoside kinase	4
ECD_02029	*yegW*	no		Putative transcriptional regulator	10
ECD_02065	*yohC*	no		Yip1 family	10
ECD_02067	*yohF*	no		Predicted oxidoreductase	10
ECD_02073	*sanA*	no		Vancomycin sensitivity	2

ECD_02090	*yeiJ(nupX)*	yes		Nucleoside permease	4
ECD_02107	*yejA*	no		Microcin C transporter YejABEF	9
ECD_02109	*yejE*	no		Microcin C transporter YejABEF	9
ECD_02145	*rcsC*	no		Regulator of capsule synthesis	9
ECD_02155	*yfaT*	no		DUF1175 family	10
ECD_02192	*menF*	no	B-95.ΔA	Isochorismate synthase	3
ECD_02286	*ypdI*	no		Colanic acid synthesis	9
ECD_02287	*yfdY*	no		Function unknown	10
ECD_02299	*yfeO*	no		Putative ion-transport protein	5
ECD_02301	*mntH*	no	B-95.ΔA	Divalent metal cation transporter	5
ECD_02308	*yfeR*	no		LysR-type transcriptional regulator	10
ECD_02362	*yffB*	no	Both	Upstream of *dapE*	10
ECD_02388	*hda*	no	Both	DnaA regulatory inactivator	1
ECD_02396	*yfgG*	no		Function unknown	10
ECD_02411	*pbpC*	no		Penicillin-binding protein 1C	2
ECD_02435	*yphA*	no		Predicted membrane protein	10
ECD_02454	*yfhB(pgpC)*	no	Both	Phosphatidylglycerophosphatase C	2
ECD_02481	*kgtP*	no		alpha-Ketoglutarate permease	3
ECD_02486	*raiA(yfiA)*	no	Both	Ribosome-associated inhibitor A	8
ECD_02512	*ybl116*	yes		RepB plasmid partition	11
ECD_02515	*ygaT(csiD)*	yes	Both	Carbon starvation induced	8
ECD_02518	*gabT*	no	Both	4-aminobutyrate aminotransferase	5
ECD_02521	*ygaU*	no		BON-LysM protein	6
ECD_02528	*ygaM*	no	B-95.ΔA	Ribosome-binding protein	6
ECD_02542	*luxS(ygaG)*	no	B-95.ΔA	S-ribosylhomocysteine lyase	9
ECD_02553	*srlE*	no		Glucitol/sorbitol PTS permease	3
ECD_02561	*norW(ygbD)*	no	B-95.ΔA	NADH:flavoruberedoxin reductase	6
ECD_02567	*hycI*	no	B-95.ΔA	Hydrogenase 3 maturation protease	3
ECD_02577	*hypB*	no	B-95.ΔA	Hydrogenase nickel incorporation	3
ECD_02582	*ygbA*	no		Upregulated by nitrosative stress	6
ECD_02584	*pphB*	no	Both	Ser/Thr protein phosphatase 2	6
ECD_02611	*ygcN*	no		Predicted oxidoreductase	10
ECD_02627	*chpA(mazF)*	no		mRNA interferase toxin	8
ECD_02629	*relA*	no	B-95.ΔA	ppGpp synthase I	8
ECD_02630	*rumA(rlmD)*	no	Both	23S rRNA m(5)U1939 methyltransferase	1
ECD_02671	*ppdC*	no		Required for swarming phenotype	9

ECD_02677	*ptsP*	no	Both	Phosphotransferase system, enzyme I	5
ECD_02679	*mutH*	no	Both	Methyl-directed mismatch repair	7
ECD_02688	*ygeA*	no		Asp/Glu_racemase family	4
ECD_02691	*kduI*	no		Hexuronate utilization in high osmolarity	3
ECD_02692	*yqeF*	no		Acetyl-CoA acetyltransferase homolog	3
ECD_02694	*yqeH*	yes		Part of T3SS PAI ETT2 remnant	9
ECD_02696	*insF-5*	yes		*IS3* transposase B	11
ECD_02709	*yqeC*	no		Function unknown	10
ECD_02710	*ygfJ(mocA)*	no	B-95.ΔA	CTP:molybdopterin cytidylyltransferase	4
ECD_02724	*recJ*	no	Both	ssDNA 5'-3' exonuclease	1
ECD_02754	*argO(yggA)*	no		Arginine exporter	4
ECD_02783	*yggU*	no		Widely conserved ACR protein	10
ECD_02791	*mutY*	no	Both	G-A mismatched DNA repair	7
ECD_02825	*ybl129*	yes		Pyrophosphorylase	10
ECD_02849	*glcC*	no		Transcriptional activator for *glc* operon	3
ECD_02858	*ybl138*	yes		Function unknown	10
ECD_02862	*yghT*	no		Predicted ATP-binding protein	10
ECD_02899	*ygiZ*	no		Inner membrane protein	10
ECD_02905	*yqiB*	no		DUF1249 family	10
ECD_02918	*yqiI*	no		Fimbrial protein	6
ECD_02930	*ttdR(ygiP)*	no		Transcriptional activator of *ttdABT*	3
ECD_02975	*yhaK*	no		Redox-sensitive bicupin	6
ECD_02985	*tdcA*	no	B-95.ΔA	Transcriptional activator of *tdc* operon	4
ECD_03017	*yraP(ecfH)*	no	B-95.ΔA	Outer membrane lipoprotein	2
ECD_03027	*yhbW*	no		Multicopy suppressor of D*rpoE*	6
ECD_03030	*nlpI*	no	Both	Lipoprotein	2
ECD_03047	*dacB*	no		Penicillin-binding protein 4	2
ECD_03069	*ptsN*	no	Both	PTS IIA-like nitrogen-regulatory protein	5
ECD_03076	*yhcC*	no		Radical SAM superfamily	10
ECD_03099	*yhcO*	no		Predicted barnase inhibitor	10
ECD_03109	*mreC*	no	Both	Cell shape-determining	2
ECD_03144	*yhdN*	no		Heat shock protein	6
B21_03152	*yheV*	no		Function unknown	10
ECD_03201	*kefB*	no	B-95.ΔA	NEM-activatable K+/H+ antiporter	5

ECD_03224	*frlD(yhfQ)*	no		Fructoselysine 6-kinase	3
ECD_03264	*bioH*	no	B-95.ΔA	Pimeloyl-ACP methyl ester esterase	4
ECD_03265	*gntX(yhgH)*	no	Both	Required for stationary phase competition	3
ECD_03268	*malQ*	no		Amylomaltase	3
ECD_03281	*glgA*	no	Both	Glycogen synthase	3
ECD_03294	*yrhA*	no		Function unknown	10
ECD_03298	*ugpQ*	no		Glycerophosphodiester phosphodiesterase	8
ECD_03360	*gadE(yhiE)*	no	B-95.ΔA	Acid-responsive regulator of *gadABC*	6
ECD_03364	*gadX*	no	B-95.ΔA	Acid-responsive regulator of *gadABC*	6
ECD_03377	*yhjK*	no	Both	Cyclic-di-GMP phosphodiesterase	8
ECD_03383	*yhjR*	no		Conserved protein	10
ECD_03405	*yiaG*	no		Predicted transcriptional regulator	10
ECD_03421	*xylR*	no		Transcriptional activator for *xylAB*	3
ECD_03434	*sgbU*	no		Putative hexulose-6-phosphate isomerase	3
ECD_03436	*ybl147*	yes		AraC family	10
ECD_03448	*rhsA*	no		RhsA element core protein	9
ECD_03449	*yibA*	no		Lyase containing heat-repeat protein	9
ECD_03450	*yibJ*	no		Predicted Rhs-family protein	9
ECD_03463	*lldD(lctD)*	no		l-lactate dehydrogenase	3
ECD_03464	*yibK(trmL)*	no	Both	tRNA (Leu) methyltransferase	1
ECD_03473	*waaH(yibD)*	no		Glucuronic acid transferase	9
ECD_03480	*waaV(ybl153)*	yes		Putative beta1,3-glucosyltransferase	9
ECD_03491	*coaD*	no	Both	Phosphopantetheine adenylyltransferase	4
ECD_03502	*dinD*	no		Damage inducible	6
ECD_03504	*ligB(yicF)*	no		DNA ligase B	1
ECD_03517	*ybl161*	yes		ShiA-like protein	10
ECD_03524	*aec66*	yes		transposase	11
ECD_03525	*ybl166*	yes		Function unknown	10
ECD_03540	*aec79*	yes		Function unknown	10
ECD_03542	*setC(yicK)*	no		Putative arabinose efflux transporter	3
ECD_03548	*yicO*	no		Predicted adenine permease	4
ECD_03556	*ivbL*	no		*ilvB* operon leader peptide	4
ECD_03560	*yidH*	no		DUF202 family	6
ECD_03573	*cbrA(yidS)*	no		Colicin M resistance protein	8

ECD_03599	*yieH*	no	Both	6-phosphogluconate phosphatase	3
ECD_03601	*cbrC(yieJ)*	no		Required for colicin E2 tolerance	8
ECD_03621	*atpE*	no	Both	ATP synthase subunit c	3

Supplementary Table 2 | Modifications (shown with "after") to the genomic sequences (shown with "before") introduced by the oligonucleotide-mediated recombination. The genes are arranged in the order of the actual engineering steps. Stop codons are underlined. Changed bases are shown in red, except for the G-to-C changes introduced by C-to-C mismatches and isolated from other changes (shown in green).

---

ubiF	TAG to TAA
before	GAAATATGCGTTAGGGTTGTAGCCTTACAACATTGCCGGA
after	GAAATATGCGTTAGGGTTGTAAAAGGCCAACATTGCCGGA
sucB	TAG to TAA
before	GCGTCTGCTGCTGGACGTGTAGTAGTTTAAGTTTCACCTG
after	GCGTCTGCTGCTGGACGTGTAACGACCTAAGTTTCACCTG
hda	TAG to TAA
before	GAAAGAAATTCTGAAGTTGTAGATAAGGTGTTTATTG*TCG*
after	GAAAGAAATTCTGAAGTTGTAAGCGGTGTGTTTATTG*TCG*
note	A putative terminator is written in italic letters.
lpxK	TAG to TAA
before	CTTGCTGGCTTCTGGCAACTAGTTACGCCGCGGCAGCGTT
after	CTTGCTGGCTTCTGGCAACTAAGGCATCCGCGGCAGCGTT
coaD	TAG to TGA
before	GCTGATGGCGAAGTTAGCGTAGCGTTTA*TGCCGGATGGTA*
after	GCTGATGGCGAAGTTAGCCTGAACTTTA*TGCCGGATGGTA*
note	REPt274 is written in italic letters.
raiA	TAG to TAA
before	CGAAGAAGTTGAAGAAGAGTAGTCCTTTATATTGAGTGTA
after	CGAAGAAGTTGAAGAAGAGTAAAAAGGTATATTGAGTGTA
mreC	TAG to TGA
before	TGCGCCGGGAGGGCAATAgtggcgagctatcgtagccagg
after	TGCGCCGGGAGGGCAGTGatggcttcttatcgtagccagg

note The *mreD* ORF is written in lowercase letters.

mutT TAG to TAA
before TGCGAAGCTTAAACGTCTGTAGGTCAGATAAGGCGTTTTC
after TGCGAAGCTTAAACGTCTGTAATGGCTATAAGGCGTTTTC

atpE TAG to TAA
before CGTGATGTTCGCTGTCGCGTAGTAAGCGTTGCTTTTATTT
after CGTGATGTTCGCTGTCGCGTAAGCCCAGTTGCTTTTATTT

mutH TAG to TAA
before CCGTCATTTTCTGATCCAGTAGCCATCGCTTTGACCTGCC
after CCGTCATTTTCTGATCCAGTAAAACGAGCTTTGACCTGCC

mutY TAG to TAA
before ACGCACTGGCGCGCCGGTTTAGCGCGTGAGTCGATAAAGA
after ACGCACTGGCGCGCCGGTTTAAACACGGAGTCGATAAAGA

ptsP TAG to TAA
before GCTGATTCGCGGAGGGTTATAGCGCGGATCATATACATAT
after GCTGATTCGCGGAGGGTTATAAGTGTTATCATATACATAT

ulaF TAG to TAA
before CGCTTATTACGGGCAGAAGTAGAACACGCGCTGCGGAAAT
after CGCTTATTACGGGCAGAAGTAACCAGACCGCTGCGGAAAT

pgpC TAG to TAA
before TGAACTCCAGCAACTGGAATAGAGTAAAGCATAGCGTCCG
after TGAACTCCAGCAACTGGAATAATTGCCAGCATAGCGTCCG

ptsN TAG to TAA
before AGGTACTCCGGATGAAGCGTAGTTATTCGGTAATGTCTCT
after AGGTACTCCGGATGAAGCGTAAGGCACCGGTAATGTCTCT

fabH TAG to TGA
before CTCCGCGCTGGTTCGTTTCTAGGATAAGGATTAAAACATG

after CTCCGCGCTGGTTCGTTTCTGATGAGAGGATTAAAACATG

pgpA TAG to TAA
before GCCGCTGGGCATTCTGTCGTAGTTGTGCACCGAT*GCCT*
after GCCGCTGGGCATTCTGTCGTAAGGTGTCACCGAT*GCCT*
note REP38 is written in italic letters.

yhjK TAG to TAA
before GAGTTACCTGGAAGAAAAGTAGCTACCCCAAACTGATTAC
after GAGTTACCTGGAAGAAAAGTAAGGTGGCCAAACTGATTAC

nlpI TAG to TAA
before GGCAGAATCGGACCAGCAATAGCTGACGTACACATCAGCC
after GGCAGAATCGGACCAGCAATAAGAACGGTACACATCAGCC

cspD TAG to TAA
before AGTAGAAGCGGCAGTCGCATAGCTCTTCTGTCTCATTGTG
after AGTAGAAGCGGCAGTCGCATAAACACCCTGTCTCATTGTG

yihW TAG to TAA
before TCTTCCCCAGGAAGACGCGTAGATTTTTGTGACCGTTAAC
after TCTTCCCCAGGAAGACGCGTAATGGAGTGTGACCGTTAAC

dosC TAG to TAA
before ACTCTGGAAAGCCAGTCTTTAGATGCGCCAGGATGCAGAG
after ACTCTGGAAAGCCAGTCTTTAACACACCCAGGATGCAGAG
note The −35 and −10 elements of the transcription promoter for *dosP* are enclosed.

yajL TAG to TAA by 8-base insertion
before TATTACGAGTAGT*GTCGGATGCGGCAAACGTCGC*
after TATTACGAGTAATCGAGTAGT*GTCGGATGCGGCAAACGTCGC*
note REP39 is written in italic letters.

glgA TAG to TAA
before GCTTGAAATAGTTTTCAGGAAACGCCTATatgaatgctcc
after GCTTGAAATAAACACGAGGAAACGCCTATatgaatgctcc

note Part of the *glgP* ORF is indicated with lowercase letters.

yibK TAG to TAA
before GGGAGCGTTGTTGAGAGATTAGTTACTGTATGCCGGATAG
after GGGAGCGTTGTTGAGAGATTAAGACGCGTATGCCGGATAG

priC TAG to TGA
before GTTTAACCCGCTAGCAATGGAGAGAATatgtcactggaaa
after GTTTAACCCGCTGAACCAGGAGAGAATatgtcactggaaa
note Part of the *ybaM* ORF is indicated with lowercase letters.

csiD TAG to TAA
before CCACTACCAGACGCATCAGTAGCGCAAAGGAATTGAGCGG
after CCACTACCAGACGCATCAGTAAATACCAGGAATTGAGCGG

priB TAG to TGA
before ATAGATTCTGGAGACTAGCCATatggcacgttatttccgt
after ATAGATTCTGGAGACTGATAGCatggcacgttatttccgt
note Part of the *rpsR* ORF is indicated with lowercase letters.

gabT TAG to TAA
before TTTTGCTGAGGCGAAGCAGTAGCTATGTCCGTTTGTAAGAGT
after TTTTGCTGAGGCGAAGCAGTAAGATGCTCCGTTTGTAAGAGT

yffB TAG to TGA
before TTTTTCCATGAGGTGTAGTCTatgtcgtgcccggttattg
after TTTTTCCATGAGGTGTGAATCatgtcgtgcccggttattg
note Part of the *dapE* ORF is indicated with lowercase letters.

creC TAG to TAA
before ACTTCACCGTCACTTCACATAGCTTCAAATTCTTCCCACA
after ACTTCACCGTCACTTCACATAATGAACAATTCTTCCCACA
note Part of the CreB binding site is enclosed.

hemY TAG to TAA
before GCAGAACAACCCGCCACAGTAGTTCTTTCTCA*CCCGGAGG*

after GCAGAACAACCCGCCACAGTAAAAGACTCTCA*CCCGGAGG*

note Part of a putative terminator is shown in italic letters.

mreC AGG to CGG

before TGCTAATCGCTCTCCACAAAGGGCTACGCCGCCGCAAAGT

after TGCTAATCGTAGCCCGCAACGGGCTACGCCGCCGCAAAGT

ligA AGG to CGT

before TCCCGACGCTGAATACGACAGGCTGATGCGCGAACTGCGC

after TCCCGACGCTGAATACGATCGTTTAATGCGCGAACTGCGC

lolA TAG to TGA

before AGATGATCAACGTAAGTAGAGGCACCTGAgtgagcaatct

after AGATGATCAACGTAAGTGAGGAGACCTGAgtgagcaatct

note Part of the *rarA* ORF is indicated with lowercase letters.

mrdB AGG to CGC

before AATGTCAATCCACACCCACAGGAAAATGTTGTCGAAAAGC

after AATGTCAATCCACACCCACCGCAAAATGTTGTCGAAAAGC

note The −10 element of the promoter for *rlpA* is enclosed.

ispE AGG to CGC

before CCCGCGCAATACGCCAAAAAGGTCAATAGAAACGTTGCTA

after CCCGCGCAATACGCCAAAACGCTCAATAGAAACGTTGCTA

murF TAG to TGA

before ACAGGAGAATGGGACatgttagtttggctggccgaacatt

after ACAGGAGAATGGGACatgctgatctggctggccgaacatt

note Part of the *mraY* ORF is indicated with lowercase letters.

note Accompanied by *mraY* (V3I)

asnS AGG to CGC

before CATTCTCGAAAACTGCGGCAGGAAGTTTGAAAACCCGGTT

after CATTCTCGAAAACTGCGGCCGCAAGTTTGAAAACCCGGTT

plsX TAG to TAA

before CAAAAGCGGAACTCTGCGGTAGCAGGACGCTGCCAGCGAA
after CAAAAGCGGAACTCTGCGGTAATCTTCAAGTGCCAGCGAAC

wzzE TAG to TGA
before AACCCGCCGTTGCTCGAAATAGCAACACTGCT*GCGGTGAG*
after AACCCGCCGTTGCTCGAAATGATTTGACTGCT*GCGGTGAG*
note Part of a putative terminator is shown in italic letters.

ispB AGG to CGC
before GGATGAATCAGATATGCGCAGGGGTAAAGCTACCGCCAAC
after GGATGAATCAGATATGCGCCGCGGTAAAGCTACCGCCAAC

pabC TAG to TGA
before TTTGTGAGCGCCCGAATTAGTCatgaaaaaagtgttattg
after TTTGTGAGCGCCCGAATTGAGAatgaaaaaagtgttattg
note Part of the *yceG* ORF is indicated with lowercase letters.

otsA TAG to TAA
before TTTCCAAAGCTTGCGTAGGAGCGGttaatctcccgtaagt
after TTTCCAAAGCTTGCGTAATCT ACGttaatctcccgtaagt
note Part of the *uspC* ORF is indicated with lowercase letters.

ydiA TAG to TGA
before TCGCCGAAT**GTACTAGAGAACTAGTGCATTAGCTT**ATTTT
after TCGCCGAAT**GTACTGATCTGCTAGTGCATTAGCTT**ATTTT
note The −35 element of the promoter for *aroH* is enclosed.
note A TrpR binding region is indicated with bold letters.

dmsD TAG to TAA
before GGTTAAACCGCTGTTTCGATAGGATCACCGTAATATT*GCC*
after GGTTAAACCGCTGTTTCGATAATTGAGCCGTAATATT*GCC*
note REPt125 is written in italic letters.

pphB TAG to TGA
before GTCATTTTATAAAATAAAGTAGT*CTCATGCTTCTTCTGTG*
after GTCATTTTATAAAATAAAGTGAA*GTCATGCTTCTTCTGTG*

note Part of a putative terminator is indicated in italic letters.

yceF TAG to TGA
before GAAAAACCCGCTGATGGGATAGTTGTTTGC*GCCGGATGTG*
after GAAAAACCCGCTGATGGGATGAGCACTTGC*GCCGGATGTG*
note REPt101b is written in italic letters.

btuF TAG to TAA
before TGCGCTTTCACAGGTAGATT*AGCGCCAGACCTTTTCAGGT*
after TGCGCTTTCACAGGTAGATT*AACCCCAGACCTTTTCAGGT*
note Part of a putative terminator is indicated in italic letters.

moaE TAG to TAA
before GCAGGCGGCAAAACGCTGGTAGTTTTTTGTTA*GCCGGATA*
after GCAGGCGGCAAAACGCTGGTAAACAACTGTTA*GCCGGATA*
note REPt74 is written in italic letters.

pssA AGG to CGC
before CGCGTTGTATGAAGCTAAAAGGCAGCGTCCGGAACTGGAT
after CGCGTTGTATGAAGCTAAACGCCAGCGTCCGGAACTGGAT

recJ TAG to TGA
before CGACAATATCTGGCCAATTTAGCGTCATCTTCTCTATAAA
after CGACAATATCTGGCCAATTTGATACGATCTTCTCTATAAA

recQ TAG to TGA
before CGATGGCGACGACGAAGAGTAGTCAGCAGCATAAAAAAGT
after CGATGGCGACGACGAAGAGTGAAATCCAGCATAAAAAAGT

nudG TAG to TAA
before cgccgccagaccagcggattagtgctaagggttttgtcat
after cgccgccagaccagcggattaagagacagggttttgtcat
note Part of the *ynjH* ORF is shown with lowercase letters.
note Accompanied by *ynjH* (T80L)

nei / abrB TAG/TAG to TAA/TAA

before GCCCTGGCTGCCAGCActaggccgaccgcttcggcgcata
after GCCCTGGCTGCCAGCAttaagcgctgcgcttcggcgcata
note Part of the *abrB* ORF is shown with lowercase letters.

rmuC TAG to TAA
before ATATAATCAGCAGTCGCGCTAGCCCATTGGGGGTAGTTAA
after ATATAATCAGCAGTCGCGCTAAGAAGCTGGGGGTAGTTAA
note Part of the promoter for *ubiE* is enclosed.

yjjX TAG to TAA
before TGCCGTATATCAGGCATTGTAGGACGGATAAGGCGTTCAC
after TGCCGTATATCAGGCATTGTAATCTACATAAGGCGTTCAC

yieH TAG to TGA
before GGGATATTACGGCATAGTTCTTCACACTCCCTTCACTTAC
after GGGATATTACGGCATGAGCATTCACACTCCCTTCACTTAC
note The −35 element of the promoter for *cbrB* is enclosed.

panE C-terminus extension
before tggtttgcctcgcccctggtagtgaagagactgaagccgt
after tggtttgcctcgcccctggctctgaagagactgaagccgt
note The new stop codon is also underlined.
note Part of the *yajL* ORF is shown with lowercase letters.

ycaI Creating a premature stop codon by deletion
before ATAAAGGAAATAACCATTCCTGTGTGGTTAAGGTTGATGA
after ATAAAGGAAATAACCAC---------AGTGGTTAAGGTTGATGA
note The premature stop codon is underlined.

baeR TAG to TGA
before CGACGCCTGCCGCATCGTTTAGTTTTAGCGACATTATTTT
after CGACGCCTGCCGCATCGTTTGAACACAGCGACATTATTTT

cpxP TAG to TAA
before TAGCAACTCACGTTCCCAGTAGTAAACCCTGTTTTCCTTG
after TAGCAACTCACGTTCCCAGTAAGTTTGCCTGTTTTCCTTG

gntX TAG to TAA

before GTGCCTTTGTCGAACCTTGTAGAGCCTCGATGATGGGCGT

after GTGCCTTTGTCGAACCTTGTAATTAAGCGATGATGGGCGT

note The −35 element of the promoter for *nfuA* is enclosed.

argR Changing Leu at position 70 to Pro correspondingly to *argR* from K-12

before GGAAATGGTTTACTGCCTGCTAGCTGAACTGGGTGTACCA

after GGAAATGGTTTACTGCCTCCCAGCTGAACTGGGTGTACCA

rumA TAG to TGA

before ACTTTTCTCGCGCGTTAAATAGTTGCGATTTGCCGATTTC

after ACTTTTCTCGCGCGTTAAATGAGATAGATTTGCCGATTTC

msrA TAG to TAA

before CTGTCTGCCACCGGAAGCATAGCGTTACGGGTACAAATGT

after CTGTCTGCCACCGGAAGCATAACCTTACGGGTACAAATGT

****

The resulting strain from the modification to *msrA* was B-59, and B-60.ΔA::Z was created by a *prfA* knockout together with the UAG replacement at the end of *hemA*.

****

speG Spontaneous 1-base deletion during recombination

before CCTGAAGCCGACCGCACAATAGCAttaataataatcgatc

after CCTGAAGCCGACCGCACA--TGATGttaataataatcgatc

note Part of the *ynfC* ORF is shown with lowercase letters.

pdhR TAG to TAA

before TCTGGAGCAACGAAAGAATTAGTGATTTTTCTGGTAAAAA

after TCTGGAGCAACGAAAGAATTAATAGCCTTTCTGGTAAAAA

ytfG TAG to TGA

before TCTTTTTAATGTTAATAAATAGTTAATTAAAGTGGCATCC

after TCTTTTTAATGTTAATAAATGAGCAATTAAAGTGGCATCC

note The −10, −24, and −35 elements of the promoter for *ytfF* are enclosed.

menF TAG to TAA
before TACTTTATTACAAATGGAATAGTAATGAGTCGCATCATTA
after TACTTTATTACAAATGGAATAAGCTCCAGTCGCATCATTA

ilvA TAG to TGA
before CAGGTTCTTTTTGGCGGGTTAGGGAAAAAT*GCCTGATAGC*
after CAGGTTCTTTTTGGCGGGTTGATTCTAAAT*GCCTGATAGC*
note REP285 is written in italic letters.

ycjU TAG to TGA
before GGCCTTCTGGCAAAACGTATAGCAAAGGAATCAACatggc
after GGCCTTCTGGCAAAACGTATGATTAAGGAATCAACatggc
note Part of the *ycjV* ORF is shown with lowercase letters.

gadE TAG to TAA
before GGGTATCACATCTTATTTTTAGTCAGGACATAAGCAACTG
after GGGTATCACATCTTATTTTTAAGTTCCACATAAGCAACTG

gadX TAG to TAA
before TGCGGAAGGAATAAGATTATAGAGTTTTACTCAGACATAA
after TGCGGAAGGAATAAGATTATAATCCAGTACTCAGACATAA

bioD TAG to TAA
before CATAAACCTTGCCTTGTTGTAGCCATTCTGTATTTGGTTA
after CATAAACCTTGCCTTGTTGTAATGCAGCTGTATTTGGTTA

bioH TAG to TAA
before GGCGTTGAAGCAGAGGGTGTAGGCAACTTTTGAAA*TGGCG*
after GGCGTTGAAGCAGAGGGTGTAATGTTGTTTTGAAA*TGGCG*
note Part of a putative terminator is indicated in italic letters.

speG Fixing a spontaneous frameshift mutation by a 1-base insertion
before CCTGAAGCCGACCGCACA--TGATGttaataataatcgatc
after CCTGAAGCCGACCGCACAATAACAttaataataatcgatc
note TGA was changed to TAA.

note Part of the *ynfC* ORF is shown with lowercase letters.

nlpC TAG to TGA

before CTGGCAGGCGAGACGAATCTAGTATGTCGAAAAA*TGGACG*

after CTGGCAGGCGAGACGAATCTGAAGACTCGAAAAA*TGGACG*

note Part of a putative terminator is written in italic letters.

ydcL TAG to TGA

before GTTTGACGTTAACAAAAAGTAGTTCCAGAC*GCGCCATCGT*

after GTTTGACGTTAACAAAAAGTGAAGGTGGAC*GCGCCATCGT*

note Part of a putative terminator is written in italic letters.

ytfJ TAG to TAA by 11-base insertion

before TTAAttaataaatagacactctgaatcccgg

after TTAATTAATAAATAAttagtagatgctcactctgaatcccgg

note Part of the *cysQ* ORF is shown with lowercase letters.

hypB TAG to TAA by 10-base insertion

before GGAGACACAGCGatgtgcataggcgttccc

after GGAGACACAGAGATGCGCGTAAatgtgcataggcgttccc

note Part of the *hypC* ORF is shown with lowercase letters.

hycI TAG to TGA

before GTTAGCGGTGGAAGAAGAGTAGTTTTTCAATAAGGAATCA

after GTTAGCGGTGGAAGAAGAGTGAGACATCAATAAGGAATCA

plsX(D14G) Changing Gly at position 14 to Asp

before GTTAGATGTCATGGGAGGGGGTTTTGGCCCTTCCGTGACA

after GTTAGATGTCATGGGAGGTGACTTTGGCCCTTCCGTGACA

thiQ TAG to TGA

before ACTATTGGGGATTACGGGTTAGTATGCACT*GTCAGATGCG*

after ACTATTGGGGATTACGGGTTGAACCACACT*GTCAGATGCG*

note REP7b is written in italic letters.

luxS TAG to TAA

before GTTGCAGGAACTGCACATCTAGTCAGTAAACTATCTTCAC

after GTTGCAGGAACTGCACATCTAAGTTACAAACTATCTTCAC

serA(F277L) Changing Leu at position 277 to phenylalanine

before GGCGACCAATAGCGATCCATTAACCTCTCCGCTGTGTGAA

after GGCGACCAATAGCGATCCATTCACTAGCCCGCTGTGTGAA

rimK TAG to TGA

before TTGCCTGAAAACGGGTGGTTAGTCGCAATCACATTACTGA

after TTGCCTGAAAACGGGTGGTTGAGAAAGATCACATTACTGA

mocA TAG to TAA

before GAAAAGGTATACTGAAATTTAGAAAATGTAGTTAAACGAT

after GAAAAGGTATACTGAAATTTAATCTGCGTAGTTAAACGAT

norW TAG to TAA

before GTTGAAAACATTGCCGATGTAGGTGGGCTACTGTGCCTAA

after GTTGAAAACATTGCCGATGTAATCATCCTACTGTGCCTAA

tdcA TAG to TAA

before GCAATTAATAGAAGTTGGTTAGTTATTTGTTTTTATTTAA

after GCAATTAATAGAAGTTGGTTAAAGCACTGTTTTTATTTAA

ycgX TAG to TAA

before AAATAATAAACCATTAATCTAGCTAAAGTTGGATGCTTAA

after AAATAATAAACCATTAATCTAATCGTCGTTGGATGCTTAA

btuC TAG to TGA

before ATTGTTAAAAGCAGGACGTTAGCCGCAAAAAGACGGTCTA

after ATTGTTAAAAGCAGGACGTTGATTCGGAAAAGACGGTCTA

tatD TAG to TAA by a 6-base insertion

before GTTTGGGATTGCGTTttagagtttgcggaactcg

after GTTTGGGATTGCGTTCTAATCttagagtttgcggaactcg

note Part of the *rfaH* ORF is shown with lowercase letters.

asmA	TAG to TAA
before	GAAGTTGCTGGAGAAGATGTAGGTTACCAGGTGACCTGTC
after	GAAGTTGCTGGAGAAGATGTAATCCTGCAGGTGACCTGTC
relA	TAG to TAA
before	GCGTCGGTTGCACGGGAGTTAGGCCGAAATTTGCTCGTAT
after	GCGTCGGTTGCACGGGAGTTAATTACTTCTTTGCTCGTAT
note	A Fis binding site for *mazEFG* is enclosed.
gsiD	TAG to TGA
before	GGATCCGAAAATTAAAGGATAGTTACGTTTGAATATTGCT
after	GGATCCGAAAATTAAAGGATGAAGGAGTTTGAATATTGCT
mntH	TAG to TGA
before	GGGTACGGCACTGGGATTGTAGTTGAATGAGCGTCGCATC
after	GGGTACGGCACTGGGCCTCTGAGTGAATGAGCGTCGCATC
kefB	TAG to TGA
before	CGGCTGGGATGAATTTGAGTAGAGGGTAAAGATGGCAATC
after	CGGCTGGGATGAATTTGAGTGATAACAAAGATGGCAATC
ycbX	TAG to TGA by 6-base insertion
before	CTTAAGTTGGCGCGttagactgcctgttcgaggc
after	CTTAAGTTGGCGCGTTGAGCttagactgcctgttcgaggc
note	Part of the *ycbW* ORF is shown with lowercase letters.
rlpA	TAG to TGA
before	ATTTATTACCACCGCGCAGTAGCGTAAAGCAGGCATCTGA
after	ATTTATTACCACCGCGCAGTGATAACAAGCAGGCATCTGA
ybjK	TAG to TAA
before	GGTTGAGAGGGTTGCAGGGTAGTAGATAAGTTTTAGAT*AA*
after	GGTTGAGAGGGTTGCAGGGTAAACTCCAAGTTTTAGAT*AA*
note	Part of the *rybB* sRNA is written in italic letters.
ycjZ	TAG to TAA

before GAAGTATAAAGGTGCTGTTTAGCACTACTTGCTGATACAT

after GAAGTATAAAGGTGCTGTTTAATGTGGCTTGCTGATACAT

yraP TAG to TGA

before AGCGTTTACGTTTATTAAATAGCAGCCCCTTGTAATG*CCT*

after AGCGTTTACGTTTATTAAATGATCTGCCCTTGTAATG*CCT*

note REP240a is written in italic letters.

ygaM TAG to TAA

before GTTAAGCATGCGCAAATCGTAGTGCAAAAATGATAATAAA

after GTTAAGCATGCGCAAATCGTAAATTGGAAATGATAATAAA

****

The resulting strain from the modification to *ygaM* was B-94.

****

hemA-prfA-hemK *hemA*(TAG to TAA) and Δ*prfA*

before TCGGGCTGGAGTAG———*prfA*—ATGGAATATCAA

after TCGGGCTGGAGTAAccaaaggatacacaATGGAATATCAA

note The start codon of *hemK* is also underlined.

Supplementary Table 3 | Spontaneous mutations occurring in BL21(DE3) (a), B-59 pBeta (b), B-60.ΔA::Z (c), B-94 (d), B-95.ΔA (e), and B-95.ΔAΔ*fabR* (f) as revealed by whole-genome sequencing. Note that repeated DNA sequences including rDNA, insertion elements, and *rhs* genes were not fully mapped in the analyses.

					Found in					
Annotation	Position	Type	Reference	Sequenced	a	b	c	d	e	f
acrB(I65I)	453255	Substitution	G	A	+	+	+	+	+	+
flgF(A19V)	1136454	Substitution	C	T	+	+	+	+	+	+
yebT(V66V)	1863432	Substitution	T	C	+	+	+	+	+	+
vioA(A338V)	2006172	Substitution	G	A	+	+	+	+	+	+
yicI(H304L)	3701104	Substitution	T	G	+	+	+	+	+	+
yjiPQ(L576L)	4478852	Substitution	T	A	+	+	+	+	+	+
cdaR(L52L)	185461	Substitution	G	A		+	+	+	+	+
upstream of *sulA*	1026026	Substitution	T	C		+	+	+	+	+
terminator of *pspF*	1353260	Substitution	G	A		+	+	+	+	+
dcp(ΔA)	1575919	Deletion	T			+	+	+	+	+
yfbS(L39L)	2298490	Substitution	A	G		+	+	+	+	+
hemX (P389PAP)	3878170-1	Insertion		GGTGCA		+	+	+	+	+
plsX(D14G)	1150228	Substitution	A	G		+	+			
serA(F277L)	2888438	Substitution	A	T		+	+			
marR(R94H)	1568696	Substitution	G	A			+			
tolQ(L130L)	734203	Substitution	C	T				+	+	+
minE(Q67P)	1212505	Substitution	T	G				+	+	+
yjhB(ΔT)	4415207	Deletion	T					+	+	+
ydgF::insAB	1618674-65	IS1 transposition								+
upstream of gntT	3406753	Substitution	C	T						+
fabR(ΔA)	4069686	Deletion	A							+

Supplementary Table 4 | *E. coli* strains used in this study.

Strain	Genotype	Source
BL21(DE3)	F– *ompT gal dcm lon hsdSB*(rB- mB-) λ(DE3 [*lacI lacUV5*-T7 gene 1 *ind1 sam7 nin5*])	Novagen
B-59	BL21(DE3) derivative, 59 less TAG codons, plasmid-free	This study
B-60.ΔA::Z	B-59 derivative, *hemA*(TAG->TAA), *prfA::zeo*, plasmid-free	This study
B-94	BL21(DE3) derivative, 94 less TAG codons, plasmid-free	This study
B-95.ΔA	B-94 derivative, *hemA*(TAG->TAA), Δ*prfA*, plasmid-free	This study
B-95.ΔAΔ*fabR*	B-95.ΔA derivative, Δ*fabR*	This study

Supplementary Table 5 | Protein spots showing higher intensities in BL21(DE3) than B-94.

Group ID	BL21 (%Volume)	B-94 (%Volume)	Sum of %Volume	Difference (B-94/BL21)
188	0.06	N.D	0.06	N/A
164	0.06	N.D	0.06	N/A
942	0.14	0.04	0.18	0.29
1633	0.11	0.04	0.15	0.36
474	0.06	0.03	0.09	0.43
1568	1.99	0.90	2.89	0.45
543	0.17	0.09	0.25	0.51
1206	0.16	0.09	0.24	0.55
870	0.08	0.04	0.12	0.56
1136	0.20	0.12	0.32	0.59
788	0.08	0.05	0.12	0.61

Supplementary Table 6 | Protein spots showing higher intensities in B-94 than BL21(DE3).

Group ID	BL21 (%Volume)	B-94 (%Volume)	Sum of %Volume	Difference (B-94/BL21)
1321	N.D	0.05	0.05	N/A
1385	0.16	0.38	0.53	2.40
1111	0.04	0.09	0.12	2.38
874	0.45	0.79	1.24	1.74
1231	0.04	0.07	0.11	1.60
776	0.12	0.19	0.31	1.53

Supplementary Table 7 | Protein spots showing higher intensities in BL21(DE3) than B-95.ΔA.

Group ID	BL21 (%Volume)	B-95 (%Volume)	Sum of %Volume	Difference (B-95/BL21)
188	0.06	N.D	0.06	N/A
164	0.06	N.D	0.06	N/A
884	0.05	N.D	0.05	N/A
1023	0.11	N.D	0.11	N/A
942	0.14	0.03	0.17	0.25
798	0.17	0.06	0.23	0.34
1633	0.11	0.04	0.15	0.36
474	0.06	0.03	0.09	0.45
770	0.05	0.03	0.08	0.51
870	0.08	0.04	0.12	0.52
1570	0.06	0.03	0.09	0.52
819	0.06	0.03	0.09	0.52
933	0.05	0.03	0.07	0.56
715	0.09	0.05	0.14	0.58
1128	0.12	0.07	0.20	0.61
792	0.05	0.03	0.08	0.61
1297	0.04	0.03	0.07	0.61
1217	0.35	0.23	0.58	0.65
1206	0.16	0.10	0.26	0.66
1551	0.08	0.06	0.14	0.66

Supplementary Table 8 | Protein spots showing higher intensities in B-95.ΔA than BL21(DE3).

Group ID	BL21 (%Volume)	B-95 (%Volume)	Sum of %Volume	Difference (B-95/BL21)
1321	N.D	0.31	0.31	N/A
568	N.D	0.13	0.13	N/A
1143	N.D	0.13	0.13	N/A
1507	N.D	0.07	0.07	N/A
853	N.D	0.06	0.06	N/A
882	0.02	0.24	0.26	10.29
1155	0.03	0.24	0.27	7.92
511	0.08	0.40	0.48	5.31
1157	0.04	0.21	0.25	5.06
1111	0.04	0.12	0.15	3.17
1087	0.02	0.05	0.07	2.82
1501	0.02	0.05	0.07	2.61
1385	0.16	0.37	0.53	2.38
1503	0.10	0.24	0.34	2.33
606	0.26	0.53	0.79	2.08
1504	0.05	0.10	0.15	2.01
1427	0.03	0.05	0.08	1.88
1221	0.14	0.25	0.39	1.80
1038	0.11	0.18	0.29	1.69
1158	0.03	0.06	0.09	1.67
930	0.07	0.11	0.18	1.62
991	0.14	0.21	0.34	1.51

Supplementary Table 9 | Protein spots identified by MS/MS ions search.

Spot numbers	Annotation
511 and 568	YfiD
884	Ybl129
1023	YhbW
1507	ClpA and GlgB
882	FrmB
1155	FrmA
606	RpsF (fully glutaminated by RimK)
188	ArgI

Supplementary Table 10 | Plasmids used in this study.

Plasmids	Parent	Replication origin	Genes	markers	Source
pMW118		pSC101	*lacZ*a	amp	Nippon Gene
pBeta	pMW118	pSC101	*lacI*q, P*tac*-*bet*-T*rrnB*	kan, amp	This study
pRF0q3	pBeta	pSC101	*lacI*q, P*tac*-*bet*-T*rrnB*, *cat*(13×Am), *supE3*	kan, tet	This study
pMINIqY	pBeta	pSC101	P*tyrT*-ile2-arg3-pro2-arg4(UCG)-*metT*-*leuW*-arg4-arg5-T*rrnC*, *lacI*q, *tetA*-*lysY*	tet	This study
pAp105		ColIb-P9 *repZ*(TAG)		kan	Ohtake *et al*., 2012
pAp15	pAp105	ColIb-P9 *repZ*(TAG)	T*rrnC*	kan	This study
pAp15-glnX	pAp15	ColIb-P9 *repZ*(TAG)	*metT-leuW-glnUW-metU-glnV-glnX*	kan	This study
pAp15-supE44	pAp15	ColIb-P9 *repZ*(TAG)	*metT-leuW-glnUW-metU-glnV-supE44*	kan	This study
pAp15-supE3	pAp15	ColIb-P9 *repZ*(TAG)	*metT-leuW-glnUW-metU-glnV-supE3*	kan	This study
pAp15-RF1	pAp15	ColIb-P9 *repZ*(TAG)	*hemA-prfA-hemK*	kan	This study
pET-sucB	pET21b(+)	pET	FLAG-*sucB*(TAG)-T7-6His	amp	This study
pET-SUMO-HV1(63Y)	pET21b(+)	pET	6His-SUMO-HV1(63Y), SfYN3	amp	This study
pET-SUMO-HV1(63Am)	pET21b(+)	pET	6His-SUMO-HV1(63Am), SfYN3	amp	This study
pET-pelB-SUMO-HV1(63Y)	pET21b(+)	pET	*pelB* leader-6His-SUMO -HV1(63Y), SfYN3	amp	This study
pET-pelB-SUMO-HV1(63Am)	pET21b(+)	pET	*pelB* leader-6His-SUMO -HV1(63Am), SfYN3	amp	This study
pET-Fab	pET26b(+)	pET	*pelB* leader-VHCH1 and *pelB*-VLCL, AzFA1	kan	This study
pET-Fab(A121X)	pET26b(+)	pET	*pelB* leader-VHCH1(A121X) and *pelB*-VLCL, AzFA1	kan	This study
pET-Fab(3×Am)	pET26b(+)	pET	*pelB* leader-VHCH1(3×Am) and *pelB*-VLCL, AzFA1	kan	This study

Supplementary Table 11 | Oligonucleotides used for the genome engineering. Lowercase letters indicate phosphorothioate moieties in the oligonucleotides.

ubiF	ccgaCCTACGGTTGGCACGCATCCGGCAATGTTGGCCTTTTACAACCCTAACGCATATTTCAGCGCCTGACGTTT
sucB	atgcCTTATCCGGTCTACAGTGCAGGTGAAACTTAGGTCGTTACACGTCCAGCAGCAGACGCGTCGGATCTTCCA
hda	gataAGGCGTTCGCGCCGCATCCGACAATAAACACACCGCTTACAACTTCAGAATTTCTTTCACAAACGGAATGG
lpxK	acatTCATGACTCCATCAATCGAACGCTGCCGCGGATGCCTTAGTTGCCAGAAGCCAGCAAGGTTAGTTGCGTAA
coaD	cccaTTTCCTGCCGGAGAATGTCCATCAGGCGCTGATGGCGAAGTTAGCCTGAACTTTATGCCGGATGGTATGCC
raiA	tgaaAGACGCCAACTTCGTCGAAGAAGTTGAAGAAGAGTAAAAAGGTATATTGAGTGTATCGCCAACGCGCCTTC
mreC	agagCCAGATTACCCAGCGTCCCTGGCTACGATAAGAAGCCATCACTGCCCTCCCGGCGCACGCGCAGGCGGTTG
mutT	gtgtGCGAATGTCGGATGCGGCGAAAACGCCTTATAGCCATTACAGACGTTTAAGCTTCGCAATTACCGGTTCAT
atpE	gttaACGTTCTGATATTGCTCTTTAAATAAAAGCAACTGGGCTTACGCGACAGCGAACATCACGTACAGACCCAG
mutH	ctacTGGCCCGTCATTTTCTGATCCAGTAAAACGAGCTTTGACCTGCCGCTTTCCGGGCATATAATTACCGCTTC
mutY	ttgtTACAGCAGTTACGCACTGGCGCGCCGGTTTAAACACGGAGTCGATAAAGAGGATGATTTATGAGCAGAACG
ptsP	ggttGCCGGATACCGTTAAAAGATATGTATATGATAACACTTATAACCCTCCGCGAATCAGCCCGCCCATGCCGC
ulaF	cagtTATCTCCCGAGGAAGGAAATTTCCGCAGCGGTCTGGTTACTTCTGCCCGTAATAAGCGTTAGGACCGTGTT
pgpC	aaaaGCGCGGCGCATTATACACGGACGCTATGCTGGCAATTATTCCAGTTGCTGGAGTTCACCGCGCGGGGTTAC
ptsN	ttacGGATACCGAAGGTACTCCGGATGAAGCGTAAGGCACCGGTAATGTCTCTTTTAGACGTTGTGAGGAGAAAC
fabH	ggaaCACAAATGCAAATTGCGTCATGTTTTAATCCTCTCATCAGAAACGAACCAGCGCGGAGCCCCAGGTGAATC
pgpA	gtcgTAGGATTTAAATAAGAGTCCAGGCCTGATGAGACGCAACAAGCGTCACATCAGGCATCGGTGACACCTTACGACAGAATGCCCAGC

yhjK	accaGCACTTTTTAAAGTTTTGTAATCAGTTTGGCCACCTTACTTTTCTTCCAGGTA ACTCTCTTCGAAGATTTC
nlpI	gatgGCAATCAAAAAAGATTACGGGCTGATGTGTACCGTTCTTATTGCTGGTCCG ATTCTGCCAGGTCATCTTGG
cspD	ttgtGCCCGTCGAAGTAGAAGCGGCAGTCGCATAAACACCCTGTCTCATTGTGTAC ATCCTAAAGGCAAAATGCC
yihW	tgtgGCAAGGTATGAAGCCAGGTTAACGGTCACACTCCATTACGCGTCTTCCTGGG GAAGAATGATTTCAATTTG
dosC	gaaaCCGTGTTGAACTCTGGAAAGCCAGTCTTTAACACACCCAGGATGCAGAGGT AATCATGAAGCTAACCGATG
yajL	aaagCCCATGAAGTGGCATCACAACTGGTGATGGCGGCAGGGATTTATAATTATT ACGAGTAATCGAGTAGTGTCGGATGCGGCAAACGT
glgA	gatgAATATGTAAACGGAGCATTCATATAGGCGTTTCCTCGTGTTTATTTCAAGCG ATAGTAAAGCTCACGGTACGACTTCGCCGCGACC
yibK	ggtgTATGAAGCCTGGCGGCAGCTGGGATATCCGGGAGCGTTGTTGAGAGATTAA GACGCGTATGCCGGATAGATGATTACATCGCATCC
priC	aatcGAAAACAGGTTAGCGCGTTTAACCCGCTGAACCAGGAGAGAATATGTCACT GGAAAATGCCCCTGACGATG
csiD	cttaCGCCACTCACCACTACCAGACGCATCAGTAAATACCAGGAATTGAGCGGAT GTATGATTTTGTGATTATTG
priB	cagaACTTGCGACGACGGAAATAACGTGCCATGCTATCAGTCTCCAGAATCTATC AATTCAATCTGCTCGGCATG
gabT	agccAGTGTTTTGCTGAGGCGAAGCAGTAAGATGCTCCGTTTGTAAGAGTTAACC GTTGCTAAGGGAATGATAAC
yffB	ccagTTATCAGCAATTTTTCCATGAGGTGTGAATCATGTCGTGCCCGGTTATTGAG CTGACACAACAGCTTATTC
creC	agcaGGATACGAAGGCTATGTGGGAAGAATTGTTCATTATGTGAAGTGACGGTGA AGTCGAAGCGAGGCCAGCAC
hemY	ccgcGACGGTTTGATGTTAACGTTGCAGAACAACCCGCCACAGTAAAAGACTCTC ACCCGGAGGCAAGCACCTCC
mreC (for AGG)	gcgcAGGCGGTTGAGCACCACTTTGCGGCGGCGTAGCCCGTTGCGGGCTACGATT AGCAGCAGGCTGTGTCGGCGCAGCAGGCGCAGTAG
ligA	cagtTCTGGATGTTTGGTTTCCAGCTCGCGCAGTTCGCGCATTAAACGATCGTATT CAGCGTCGGGAATTTCCGGCGCATCCATCACATG
lolA	ccgaAAAATCGAGCGACAGATTGCTCACTCAGGTCTCCTCACTTACGTTGATCATC TACCGTGACGCCTTGCGGC

mrdB	ctggGTTCGGGATTGTAATGTCAATCCACACCCACCGCAAAATGTTGTCGAAAAGCGTGTAAGAGGTGCGCAATG
ispE	ttaaAGATCCTGAACTCCCGCGCAATACGCCAAAACGCTCAATAGAAACGTTGCTAAAATGTGAATTCAGCAATG
murF	cggaATAATATTTGACCAAATGTTCGGCCAGCCAGATCAGCATGTCCCATTCTCCTGTAAAGCGCGTACTACCTC
asnS	tataCCGACGCAGTGACCATTCTCGAAAACTGCGGCCGCAAGTTTGAAAACCCGGTTTACTGGGGAGTCGATCTC
plsX	ccgtCACTTGCAAACTGCGAGTTCGCTGGCACTTGAAGATTACCGCAGAGTTCCGCTTTTGCCACCGTCCAGCAG
wzzE	ataaGCGGTGAGCGCCTTTGCGCTCACCGCAGCAGTCAAATCATTTCGAGCAACGGCGGGTTAATGCGACACCAG
ispB	ctacACGACGACGTTGTGGATGAATCAGATATGCGCCGCGGTAAAGCTACCGCCAACGCCGCATTTGGCAATGCC
pabC	ccagCAATAACAAGATTATCAATAACACTTTTTTCATTCTCAATTCGGGCGCTCACAAAGTGGGGCTAAATATTC
otsA	cgatGTGCTGTTAGTTCCACTTACGGGAGATTAACGTAGATTACGCAAGCTTTGGAAAGGTAGCAACTTTATCGC
ydiA	gataTCATGGGCCTTAGTCGCCGAATGTACTGATCTGCTAGTGCATTAGCTTATTTTTTTGTTATCATGCTAACC
dmsD	attcCTGTCGCGGTTAAACCGCTGTTTCGATAATTGAGCCGTAATATTGCCGGATGGTGACGATTACGCGCAATC
pphB	cgaaAAGCGGGCGGCTGTCATTTTATAAAATAAAGTGAAGTCATGCTTCTTCTGTGAAGCATGAGTAACCAAATG
yceF	tgtgTCAGATGTTACGTCGGGAAGGGAAAAACCCGCTGATGGGATGAGCACTTGCGCCGGATGTGGTTACGTCGC
btuF	gcacAACAGCTCTGTAATGCGCTTTCACAGGTAGATTAACCCCAGACCTTTTCAGGTGCTCAGTGGGGATTGAAC
moaE	gacgCAGCGGTGCCTTATCCGGCTAACAGTTGTTTACCAGCGTTTTGCCGCCTGCTGATCGCTCTCCCGAGCTTC
pssA	ggcaAAGGCATTCTGAACGCGTTGTATGAAGCTAAACGCCAGCGTCCGGAACTGGATGTGCGGGTGCTGGTCGAC
recJ	gtacCCAATCCACGCTCTTTTTTATAGAGAAGATCGTATCAAATTGGCCAGATATTGTCGATGATAATTTGCAGG
recQ	tacgGAGTCTTCATCCTGGCACTTTTTTATGCTGGATTTCACTCTTCGTCGTCGCCATCGACATGCGCACGAATC

nudG	atggCTTTACGCGCCGCCAGACCAGCGGATTAAGAGACAGGGTTTTGTCATCACGCTGGCATTGCAGCAGTATTC
nei/abrB	cgcgGTTTATTTCAACCTATGCGCCGAAGCGCAGCGCTTAATGCTGGCAGCCAGGGCACCAGTAAAACGGGCGAG
rmuC	tgccCTAGATTTCTACCCGGCTTAACTACCCCCAGCTTCTTAGCGCGACTGCTGATTATATTCATCATCGCGTTG
yjjX	tccgTTTCACAATGCCGTATATCAGGCATTGTAATCTACATAAGGCGTTCACGCCGCATCCGGCATTCGGTGCAC
yieH	actgTGGAAAGCGCGTGGTTGGGATATTACGGCATGAGCATTCACACTCCCTTCACTTACCCCGCTTAAATTGGC
panE	atgaGCGCATCGGCACTGGTTTGCCTCGCCCCTGGCTCTGAAGAGACTGAAGCCGTCACCACTATCGATCTGCTG
ycaI	gaagAATGCTATTCGTCCCGTCATCAACCTTAACCACTGTGGTTATTTCCTTTATCGTTGCTACCTTGTAAAGGC
baeR	accgCTGGGAAGCCGACGCCTGCCGCATCGTTTGAACACAGCGACATTATTTTGTTAGCCGGAGATGGCGTTCAG
cpxP	gacaGGGATGGTGTCTATGGCAAGGAAAACAGGCAAACTTACTGGGAACGTGAGTTGCTACTACTCAATAGCTTC
gntX	ctgtCCAGGTCTGGTGCCTTTGTCGAACCTTGTAATTAAGCGATGATGGGCGTATTATAACCAACTAAAATAGTC
argR	acgtACACGCAATGCCAAAATGGAAATGGTTTACTGCCTCCCAGCTGAACTGGGTGTACCAACCACCTCCAGTCC
rumA	ttagGGACCAGACCTGCCGAAATCGGCAAATCTATCTCATTTAACGCGCGAGAAAAGTACCATCGATTCCAGATG
msrA	ttggCGGAATTGGCGTCTGTCTGCCACCGGAAGCATAACCTTACGGGTACAAATGTAGATTGTTGATAAAGTGCG
speG	accgGGTCAGACTCTCCTGAAGCCGACCGCACAATGATGTTAATAATAATCGATCGTATTTTTGATGGTGTAAAC
pdhR	tttaCAACATCTTCTGGATAATTTTTACCAGAAAGGCTATTAATTCTTTCGTTGCTCCAGACGACGCAGAGAACG
ytfG	tagcCGAAAGCGTAAGCCATCTTTTTAATGTTAATAAATGAGCAATTAAAGTGGCATCCTCCCGCATCCTCTCTG
menF	ttatTGATATGAATCGGTAATGATGCGACTGGAGCTTATTCCATTTGTAATAAAGTACGCAGCCCTGCCGCTTTG
ilvA	taggCCTGATAAGCGAAGCGCTATCAGGCATTTAGAATCAACCCGCCAAAAAGAACCTGAACGCCGGGTTATTGG

ycjU	tatgTTGTAACGAAAGCTGAGCCATGTTGATTCCTTAATCATACGTTTTGCCAGAAGGCCGATAACCGCGGCCAG
gadE	catcGTCACCCTGGGTATCACATCTTATTTTTAAGTTCCACATAAGCAACTGAAATTGATGGCTGGCATGACGAG
gadX	gtccCCTATGCCGGGTTTTTTTTATGTCTGAGTACTGGATTATAATCTTATTCCTTCCGCAGAACGGTCAGTGCC
bioD	cgcgATCTCGCTCGCAATTTAACCAAATACAGCTGCATTACAACAAGGCAAGGTTTATGTACTTTCCGGTTGCCG
bioH	ccagTAAATTTCTGTCTCGCCATTTCAAAACAACATTACACCCTCTGCTTCAACGCCACCAGCAGGTGACAAAAC
speG (for repair)	accgGGTCAGACTCTCCTGAAGCCGACCGCACAATAACATTAATAATAATCGATCGTATTTTTGATGGTGTAAAC
nlpC	cggcGATGTATCATCGCCGTCCATTTTTCGAGTCTTCAGATTCGTCTCGCCTGCCAGAAATTTTTCTGCCAATAG
ydcL	ggcgCACCGGATAAAAAGCGCCATCTAACGATGGCGCGTCCACCTTCACTTTTTGTTAACGTCAAACATGGTGGC
ytfJ	agtgATGGACCTGCTGCATAAATTAATTAATAAATAATTAGTAGATGCTCACTCTGAATCCCGGATTCAGGAACG
hypB	ggacCAGTGGCTGAACTGGCTGGAGACACAGAGATGCGCGTAAATGTGCATAGGCGTTCCCGGCCAGATCCGCAC
hycI	caagAACATCCCTGTCCTGATTCCTTATTGATGTCTCACTCTTCTTCCACCGCTAACTGCGCGAAGCCGCCGTTC
plsX (for repair)	tgcaATGCTGCAGGCACTGTCACGGAAGGGCCAAAGTCACCTCCCATGACATCTAACGCCAGGGTTAGACGTGTC
thiQ	gagcGGTAAGGCGAGTGCTTCAGCACTATTGGGGATTACGGGTTGAACCACACTGTCAGATGCGGCGTGAACACC
luxS	gaacTGGCTTTTTTCAATTAATTGTGAAGATAGTTTGTAACTTAGATGTGCAGTTCCTGCAACTTCTCTTTCGGC
serA (for repair)	ggcgTCAGAAGGACGTTGTCGAATTCACACAGCGGGCTAGTGAATGGATCGCTATTGGTCGCCGGTTCCGTCGGG
rimK	cgcaGGCAAAACCATGATCAGTAATGTGATCTTTCTCAACCACCCGTTTTCAGGCAATATTCTGTCGTAGCGTGG
mocA	ctttATTACAGCGAAGAAAAGGTATACTGAAATTTAATCTGCGTAGTTAAACGATTGCGTTCAAATATTTAACCC
norW	aggcATTTGGATTGTTGAAAACATTGCCGATGTAATCATCCTACTGTGCCTAAAATGTCGGATGCGACGCTGGCG

tdcA	acagACAGGTGGATTATTTATGTTTAAATAAAAACAGTGCTTTAACCAACTTCTATTAATTGCCTTCGTCTACAC
ycgX	gaagATGTAGAAAATAATAAACCATTAATCTAATCGTCGTTGGATGCTTAAGAAATGCTTCATAATTCAGTAAGG
btuC	aaaaTCTTAGCTTTTAATCATAGACCGTCTTTTCCGAATCAACGTCCTGCTTTTAACAATAACCAGATAAACACC
tatD	gtgtGAAGAACACCGAGTTCCGCAAACTCTAAGATTAGAACGCAATCCCAAACAGTGTTTTGACATTAGCATCCG
asmA	acgcGTCTTATCAGGCCTACAGGACAGGTCACCTGCAGGATTACATCTTCTCCAGCAACTTCTTCACATCTTTGC
relA	tataTCAATCTACATTGTAGATACGAGCAAAGAAGTAATTAACTCCCGTGCAACCGACGCGCGTCGATAACATCC
gsiD	tcctGTGAGGTGATTACCCTTTCAAGCAATATTCAAACTCCTTCATCCTTTAATTTTCGGATCCAGCGCATCGCG
mntH	atccGCCAATAGTGCCAGATGCGACGCTCATTCACTCAGAGGCCCAGTGCCGTACCCACCAACAACCAGATATTC
kefB	ccgcAATAAAACGTTTTCGGATTGCCATCTTTGTTATCACTCAAATTCATCCCAGCCGTCCAGCTGGCGTCGTTC
ycbX	tagcTGTGTACCGAAGACTGCACTTAAGTTGGCGCGTTGAGCTTAGACTGCCTGTTCGAGGCTGAAGCTGTCAAC
rlpA	ccaaTTACAGTCATTTATTACCACCGCGCAGTGATAACAAGCAGGCATCTGATGTGTCAATTTCTGTAAATGACG
ybjK	tttaCATAAATGGGTTTTTTGTTATCTAAAACTTGGAGTTTACCCTGCAACCCTCTCAACCATCCTCAAAATCTC
ycjZ	gaatACGTTAAGAGAAGAATTAAATTAATGTATCAGCAAGCCACATTAAACAGCACCTTTATACTTCAGCCTATC
yraP	aaagCGGGTAACCACAGCGTTTACGTTTATTAAATGATCTGCCCTTGTAATGCCTGATGCGACGCTTGCCGCGTC
ygaM	tcggCGCACTGTTAAGCATGCGCAAATCGTAAATTGGAAATGATAATAAATACGCGTCTTTGACCCCGAAGCCTG
hemA & *ΔprfA*	cttaTTGCTTCACGTAACCAGTGTTGATATTCCATTGTGTATCCTTTGGTTACTCCAGCCCGAGGCTGTCGCGCAGAATATTCAG

Supplementary Table 12 | Primer sets used for colony-direct PCR. The numbers following the gene names indicate the expected lengths of the PCR products.

Gene, size(bp)	Forward primer	Reverse primer
ubiF, 250	CGTTAGGGTTGTAAAAGGC	CCAGTTGGGGTATCGCCAAG
sucB, 308	GCTGGACGTGTAACGACC	CATTGTCGGTCACCCGTAG
hda, 400	TTGCAGGCGATGAGTTGTGG	CGACAATAAACACACCGCT
lpxK, 200	CTTCTGGCAACTAAGGCAT	ATTGATGGTATCGATTTGCAG
coaD, 1000	AATTTATTGCTCATCCTGGTACC	CATCCGGCATAAAGTTCAG
raiA, 650	AAGAAGTTGAAGAAGAGTAAAAAGG	AGTGACGGGCAGCGTACTGAC
mreC, 800	TTCCCCGCTGCGTCAGGATG	GCTACGATAAGAAGCCATCAC
mutT, 300	AGCTTAAACGTCTGTAATGGC	AAAAACGAATCCCCAGCAGTG
atpE, 300	CACTACTACGTTTTAACTGAAACAAAC	CTTTAAATAAAAGCAACTGGGC
mutH, 200	CATTTTCTGATCCAGTAAAACG	TGCCACCACCAGAGAAAGG
mutY, 423	GCAAAAAAACCGAAACAGACG	CCTCTTTATCGACTCCGTGTT
ptsP, 400	TCTGGCCGTTGATCGCAAC	TACCGTTAAAAGATATGTATATGATA ACAC
ulaF, 400	ACGGGCAGAAGTAACCAGAC	CCTTCTGGCTGTGCTTTTGC
pgpC, 397	CAGCAACTGGAATAATTGCC	CGTCACGCGCACCAAAGAC
ptsN, 300	TCCGCATGGCAAACTGGAAG	AAAGAGACATTACCGGTGCC
fabH, 280	TCTTGACCGTTCTCAACTGG	CGTCATGTTTTAATCCTCTCATC
pgpA, 298	GCATTCTGTCGTAAGGTGTC	CAGATCGCAGAGCGGTTAAC
yhjK, 500	CCTGGAAGAAAAGTAAGGTGG	GGCTGCACGACGTTAACGATG
nlpI, 200	TCGGACCAGCAATAAGAAC	AGCCATGTAGTACGTGTGCCTC
cspD, 300	GACCCCGGTTTATTCCATCTTAC	TGTACACAATGAGACAGGGTG
yihW, 500	AGGAAGACGCGTAATGGAG	GTCAGCAGCATCACGAATTTC
dosC, 400	GGAAAGCCAGTCTTTAACACAC	GCCAGGTAATAAACTTTCCC
yajL, 300	GGGATTTATAATTATTACGAGTAATC GAG	ATATCCGTTCTGTCGATGAGCAG
glgA, 188	ACTATCGCTTGAAATAAACACG	ATCGCGCACAGCAAATAAC
yibK, 400	CGTTGTTGAGAGATTAAGACGC	GGTGAAACGGCGGTAATTG
priC, 534	GCTGCTGGAAAAACTGGAAG	GTGACATATTCTCTCCTGGTTC
csiD, 200	CCAGACGCATCAGTAAATACC	AATAAACCCCGGCATGGATC
priB, 390	TGATAGATTCTGGAGACTGATAGC	CGGTACCAGGAAGTTACGAG
gabT, 313	GGCGAAGCAGTAAGATGC	CTCGAAAGTGTGTCACTTGG
yffB, 300	TTTTCCATGAGGTGTGAATC	CGAATAACATGCCGTCACG
creC, 500	CCGTCACTTCACATAATGAAC	TAAAGGGCTTGCCTAAGATG
hemY, 200	CCCGCCACAGTAAAAGAC	TTTACGGTGCTTATCGTCCAC

mreC (for AGG), 210	GCCTGCTGCTAATCGTAGC	GATGAGTAACACCCAGTTTGG
ligA, 650	CGGAACCGGCTCCAGTTATG	CAGTTCGCGCATTAAACG
lolA, 399	GAAAGCATTCTGGTTTCTGAC	TGCTCACTCAGGTCTCCTC
mrdB, 300	GAAGAGCTGGGATTAGTGGGC	TTCGACAACATTTTGCG
ispE, 500	GACACCATCAGCATTGAGCTTC	GCAACGTTTCTATTGAGCG
murF, 500	AGAATGGGACATGCTGATC	ATGGGACCACCAGCTGCGTTG
asnS, 400	CGACAAAGACTTCTTTGGTAAAGAGTC	GGGTTTTCAAACTTGCG
plsX, 697	GAACTCTGCGGTAATCTTCAAG	TCAGATGAATTGAATTCTCTGG
wzzE, 318	GGAATTACCTGATTCAGAAATGTTC	TCACCGCAGCAGTCAAATC
ispB, 300	AATCAGATATGCGCCGC	TACAGCCAGCCAGAATTCC
pabC, 400	CAAGTATTGATTCGCTCTCATC	AAGATTATCAATAACACTTTTTTCAT TCTC
otsA, 300	CCAAAGCTTGCGTAATCTAC	CCGATGCTTGAAGATTTACG
ydiA, 300	GATATGGATAATCTGGTGCTACC	AAAAATAAGCTAATGCACTAGCAGATC
dmsD, 500	CCGCTGTTTCGATAATTGAG	GTAATCGAACTGTCCATCGG
pphB, 400	GGAAATATGTGGCTTGCCAG	TTCACAGAAGAAGCATGACTTC
yceF, 500	CCACGCAGCGACGAATCAC	ATCCGGCGCAAGTGCTC
btuF, 270	CGCTTTCACAGGTAGATTAACC	GGTGACCATTGCAACGACCAG
moaE, 400	GCAAAACGCTGGTAAACAAC	ACCTGCGCTCAGCTTTTGAATC
pssA, 300	CGTTGTATGAAGCTAAACGC	GGTCGTAGCGATATTTATCGTG
recJ, 300	AATATCTGGCCAATTTGATACG	TCGAGGGAGGAACGCTCTTTAC
recQ, 400	AAAATATTGCCCAGCATTCTGC	GCACTTTTTTATGCTGGATTTC
nudG, 500	AGACCAGCGGATTAAGAGAC	TGTCTGATCCATAGATATAAAACCC
nei / abrB, 400	TGCCAGCATTAAGCGCTG	CTGGTCAGCTCATCACCATC
rmuC, 250	AGCAGTCGCGCTAAGAAGC	GAATACCGAATGACATCAAATCATTC
yjjX, 500	CCGTATATCAGGCATTGTAATCTAC	TATCGTACTCTTTAGCGAGTACAACC
yieH, 500	CATGGTGTTACGTTAGCGAAAAC	GAAGGGAGTGTGAATGCTC
panE, 400	GCCGTCAACTGTGTGATTAATC	CGGCTTCAGTCTCTTCAGAG
ycaI, 500	GGTTCTTACTGGTGGTATGGC	CGTCATCAACCTTAACCACTG
baeR, 300	GTCGTTTTCAGGCTTCATGG	CAAAATAATGTCGCTGTGTTC
cpxP, 400	TCACGTTCCCAGTAAGTTTG	CTCAGCTTTACCGTGACCAAAC
gntX, 200	CCTTTGTCGAACCTTGTAATTAAG	AACACCACATTCAGCGTTAGG
argR, 250	AAATGGTTTACTGCCTCCC	GCTTCATACAGGTCTTTGACG
rumA, 300	AAAATCTTGAAGAAGATGTCACAAAG	CGAAATCGGCAAATCTATCTC
msrA, 400	CCACCGGAAGCATAACC	GGACATCGAGAAGAACGCAC
speG, 315	GGAGTATCAGGGGAAAGGTC	ATCAAAAATACGATCGATTATTATTAA CATC
pdhR, 300	GAGCAACGAAAGAATTAATAGCC	AGCAGTTGGTCGATCAGATACTG

ytfG, 500	ATTCTGGCATCGTTTACACC	GGATGCCACTTTAATTGCTC
menF, 227	CTTTATTACAAATGGAATAAGCTCC	GCTAACGTTAACGGTGTAGAAC
ilvA, 317	GCTACACGTGCGCTATATGG	CGCTATCAGGCATTTAGAATC
ycjU, 400	TTCTACCCGGCATTCGCTC	AGCCATGTTGATTCCTTAATC
gadE, 300	GGTATCACATCTTATTTTTAAGTTCC	ATGAGGGATTTACAGACCAGC
gadX, 400	CGGAAGGAATAAGATTATAATCCAG	AGACATGAGTCATGATTATCCC
bioD, 200	CTTGCCTTGTTGTAATGCAG	GGTTATCCACAGGAATAGTGG
bioH, 300	GAAGCAGAGGGTGTAATGTTG	ATCAAAGGGCTGTTTAATTACATAC
speG (for repair), 400	AGCCGACCGCACAATAAC	GGAAAATGCCAGCTAGCAGAATTAC
nlpC, 300	GCGAGACGAATCTGAAGAC	GCAATCAGAATGATAAAGATTCTCC
ydcL, 400	GACGTTAACAAAAAGTGAAGGTG	CACTTACTTTTCAAATGTATGTGCTG
ytfJ, 400	GAAAAGTGCGAGTACTGCAAC	GGGATTCAGAGTGAGCATCTAC
hypB, 250	AGAGATGCGCGTAAATGTG	GCTCAACGTCAAACATGTTTTG
hycI, 400	GTAACTGGGTGGTGATTGATG	GTCCTGATTCCTTATTGATGTCTC
plsX (for repair), 300	AGATGTCATGGGAGGTGAC	GTATTACCGGCACTGACACAGG
thiQ, 400	AGCAGGAGAAAATGCACGC	GCATCTGACAGTGTGGTTC
luxS, 500	CAGGAACTGCACATCTAAGTTAC	TGGCCTGCTTCTTTATGCC
serA (for repair), 250	AATAGCGATCCATTCACTAGC	TGTTCAGCGCAGTTAGCACG
rimK, 400	GAAAACGGGTGGTTGAGAAAG	AGCTTTGGCAGATTATCATCC
mocA, 400	CACTATTATTCACAACCCAGATTATG	GCAATCGTTTAACTACGCAG
norW, 250	ACATTGCCGATGTAATCATC	ACATTGCTCTTTCCACAGAG
tdcA, 515	CAATTAATAGAAGTTGGTTAAAGCAC	GTTGAAGTTATCACCATGCAG
ycgX, 500	ACAGACCAGATTGAAGTGGTAAAAC	TCTTAAGCATCCAACGACG
btuC, 200	TGTGGTTTAACCGATCATCG	AGACCGTCTTTTCCGAATC
tatD, 400	TTTGGGATTGCGTTCTAATC	AAATCGTGCGTGGAAAACGTAC
asmA, 300	GCTGGAGAAGATGTAATCCTG	ACGAGGTGTGATGGTATCGG
relA, 500	TGCACGGGAGTTAATTACTTC	GCGTTGTACAAGGAACACACAG
gsiD, 444	CATTGCTAACGTGATTATTGCAG	CAAGCAATATTCAAACTCCTTC
mntH, 300	GTTCATTCGCTTCCATATCC	ACGCTCATTCACTCAGAGG
kefB, 400	CTGGGATGAATTTGAGTGATAAC	GACTCATCAACCAACACACC
ycbX, 300	CTGATGATACCGCCAACATC	CGAACAGGCAGTCTAAGCTC
rlpA, 409	GTAACCAGCAGCGGTTTCC	ATCAGATGCCTGCTTGTTATC
ybjK, 200	GGTTGCAGGGTAAACTCC	TCCGTTAGCATGCAAATTGG
ycjZ, 200	GTATAAAGGTGCTGTTTAATGTGG	AAATGCGGTAAAGCACATAGAAC
yraP, 424	CATTGTCGAAAGACGAACAG	GGCATTACAAGGGCAGATC

ygaM, 400	ATGCGCAAATCGTAAATTGG	CTGAGCACGCAACGCTTCTG
